# The mRNA-binding Protein TTP/ZFP36 in Hepatocarcinogenesis and Hepatocellular Carcinoma

**DOI:** 10.3390/cancers11111754

**Published:** 2019-11-08

**Authors:** Tarek Kröhler, Sonja M. Kessler, Kevan Hosseini, Markus List, Ahmad Barghash, Sonika Patial, Stephan Laggai, Katja Gemperlein, Johannes Haybaeck, Rolf Müller, Volkhard Helms, Marcel H. Schulz, Jessica Hoppstädter, Perry J. Blackshear, Alexandra K. Kiemer

**Affiliations:** 1 Department of Pharmacy, Pharmaceutical Biology, Saarland University, 66123 Saarbrücken, Germany; tarek.kroehler@uni-saarland.de (T.K.); s.kessler@mx.uni-saarland.de (S.M.K.); hosseini.kevan@gmail.com (K.H.); stephanlaggai@yahoo.de (S.L.); j.hoppstaedter@mx.uni-saarland.de (J.H.); 2 Institute of Pharmacy, Department of Pharmacology for Natural Sciences, Martin Luther University Halle-Wittenberg, 06120 Halle, Germany; 3 Department for Computational Biology and Applied Algorithmics, Max Planck Institute for Informatics, Saarland Informatics Campus, 66123 Saarbrücken, Germany; markus.list@wzw.tum.de (M.L.); marcel.schulz@em.uni-frankfurt.de (M.H.S.); 4 Big Data in BioMedicine Group, Chair of Experimental Bioinformatics, TUM School of Life Sciences Weihenstephan, Technical University of Munich, 85354 Freising, Germany; 5 School of Electrical Engineering and Information Technology, German Jordanian University, Amman 11180, Jordan; ahmad.barghash@gju.edu.jo; 6 Center for Bioinformatics, Saarland University, 66123 Saarbrücken, Germany; volkhard.helms@bioinformatik.uni-saarland.de; 7 The Laboratory of Signal Transduction, National Institute of Environmental Health Sciences, Research Triangle Park, NC 27709, USA; spatial@lsu.edu (S.P.); black009@niehs.nih.gov (P.J.B.); 8 Department of Comparative Biomedical Sciences, School of Veterinary Medicine, Louisiana State University, Baton Rouge, LA 70803, USA; 9 Department of Microbial Natural Products, Helmholtz Institute for Pharmaceutical Research Saarland, Helmholtz Centre for Infection Research, 66123 Saarbrücken, Germany; KatjaGemperlein@aol.com (K.G.); rom@mx.uni-saarland.de (R.M.); 10 Department of Pathology, Medical Faculty, Otto-von-Guericke University Magdeburg, 39106 Magdeburg, Germany; johannes.haybaeck@med.ovgu.de; 11 Department of Pathology, Neuropathology and Molecular Pathology, Medical University of Innsbruck, Innsbruck 6020, Austria; 12 Institute of Pathology, Medical University of Graz, Graz 8036, Austria; 13 Cluster of Excellence in Multimodal Computing and Interaction, Saarland Informatics Campus, 66123 Saarbrücken, Germany; 14 Institute for Cardiovascular Regeneration, Goethe-University Hospital, 60590 Frankfurt, Germany; 15 German Centre for Cardiovascular Research (DZHK), Partner site Rhine-Main, 60590 Frankfurt, Germany

**Keywords:** liver cancer, NASH, chemoresistance, *NEAT1*, HepG2, Huh7, BCL2, MYC, VEGFA, flow cytometry

## Abstract

Hepatic lipid deposition and inflammation represent risk factors for hepatocellular carcinoma (HCC). The mRNA-binding protein tristetraprolin (TTP, gene name *ZFP36*) has been suggested as a tumor suppressor in several malignancies, but it increases insulin resistance. The aim of this study was to elucidate the role of TTP in hepatocarcinogenesis and HCC progression. Employing liver-specific TTP-knockout (ls*Ttp*-KO) mice in the diethylnitrosamine (DEN) hepatocarcinogenesis model, we observed a significantly reduced tumor burden compared to wild-type animals. Upon short-term DEN treatment, modelling early inflammatory processes in hepatocarcinogenesis, ls*Ttp*-KO mice exhibited a reduced monocyte/macrophage ratio as compared to wild-type mice. While short-term DEN strongly induced an abundance of saturated and poly-unsaturated hepatic fatty acids, ls*Ttp*-KO mice did not show these changes. These findings suggested anti-carcinogenic actions of TTP deletion due to effects on inflammation and metabolism. Interestingly, though, investigating effects of TTP on different hallmarks of cancer suggested tumor-suppressing actions: TTP inhibited proliferation, attenuated migration, and slightly increased chemosensitivity. In line with a tumor-suppressing activity, we observed a reduced expression of several oncogenes in TTP-overexpressing cells. Accordingly, *ZFP36* expression was downregulated in tumor tissues in three large human data sets. Taken together, this study suggests that hepatocytic TTP promotes hepatocarcinogenesis, while it shows tumor-suppressive actions during hepatic tumor progression.

## 1. Introduction

Hepatocellular carcinoma (HCC), the predominant form of liver cancer, is the second most common cause of cancer-related death worldwide [1,2]. Besides viral hepatitis in North Africa and East Asia [3], alcohol abuse, obesity, type 2 diabetes, and metabolic disorders represent the major risk factors for the development of HCC in Northern Europe, the USA, and Canada [4,5].

The initiation and progression of cancer are provoked by a dysregulated expression of proteins controlling diverse cellular phenotypes: cell cycle, differentiation, apoptosis, angiogenesis, and cell invasiveness [6]. Biosynthesis of these proteins is strongly regulated by the concentrations of their respective mRNAs in the cytoplasm, which depend on both mRNA synthesis and degradation. The cytoplasmic stability of many mRNAs is controlled by mRNA-binding proteins (RBPs), some of which have been shown to be deregulated in HCC. However, most of the studies focus on upregulated RBPs [7,8,9,10]. A subgroup of RBPs are so-called adenosine-uridine-rich (AU-rich) element binding proteins (ARE-BPs), which control the stability of mRNAs by binding to the AU-rich elements (ARE) located within their 3′-untranslated region (3′-UTR) [11]. A prominent member of these ARE-BPs, tristetraprolin (TTP, gene name *ZFP36*), accelerates the decay of transcripts [12]. TTP expression is repressed in several human cancers [13,14] and a loss of functional TTP can impact patient prognosis [15]. 

HCC usually develops in the course of metabolic changes. Recent evidence showed that hepatocytic TTP seems to rather amplify metabolic disorders by promoting insulin resistance, quite in contrast to its tumor suppressor role [16]. Since, to the best of our knowledge, nothing is known about a potential role of TTP in tumor initiation, we conducted this study to address the potential role of TTP in hepatocarcinogenesis. We analyzed the effect of a liver-specific TTP-knockout (ls*Ttp*-KO) in mice treated with the tumor-inducing agent diethylnitrosamine (DEN). In addition, we investigated the impact of TTP overexpression in a set of hallmarks of cancer in order to study cancer progression. Our findings revealed tumor-promoting actions of TTP in tumor initiation, due to metabolic and inflammatory action, but tumor-suppressive actions in HCC progression.

## 2. Results

### 2.1. TTP and Tumor Initiation

**Figure 1:**
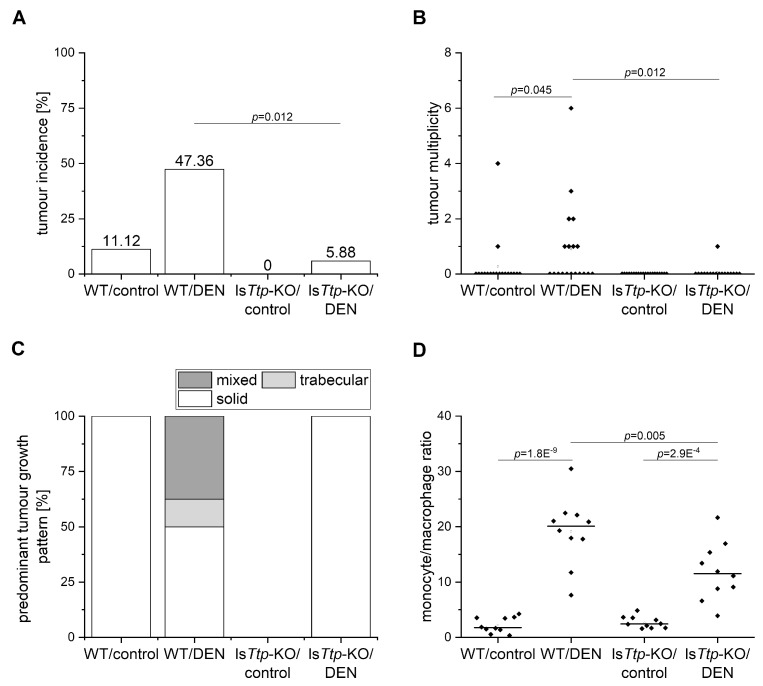
Amount and pattern of tumors and hepatic monocyte to macrophage ratio in diethylnitrosamine (DEN)- or sham-treated wild type (WT) and tristetraprolin (TTP)-knockout (ls*Ttp*-KO) mice. (**A**): Tumor incidence. (**B**): Tumor multiplicity. (**C**): Predominant tumor growth pattern. (**D**): Monocyte/macrophage ratio analyzed by multi-color flow cytometry (short-term experiment). *n* = 10, each.

In order to study the role of TTP in liver tumor initiation, we employed liver-specific *Ttp*-KO (ls*Ttp*-KO) mice [16] in the diethylnitrosamine (DEN) hepatocarcinogenesis mouse model. Wild-type and ls*Ttp*-KO mice were treated with DEN at the age of two weeks to induce tumors, and were sacrificed at the age of six months, representing an early time point and therefore rather modelling tumor initiation [17]. The tumor incidence was significantly lower in DEN-treated ls*Ttp*-KO animals compared to DEN-treated wild type animals (Figure 1A), while there was no statistical difference regarding tumor incidence between the genotypes in the sham-treated groups. The number of tumors per animal was significantly lower in the DEN-treated ls*Ttp*-KO animals compared to DEN-treated wild type mice (Figure 1B). While tumors of DEN-treated ls*Ttp*-KO mice showed a solid growth pattern, tumors of wild-type animals were of trabecular, solid, or mixed pattern (Figure 1C).

### 2.2. DEN-Induced Leukocyte Recruitment and Hepatic Lipids

Short-term (48 hh) DEN treatment represents a well-established approach to modeling metabolic and inflammatory events in early hepatocarcinogenesis [18,19]. We hypothesized that protection from DEN-induced liver cancer in ls*Ttp*-KO mice is a consequence of attenuated leukocyte recruitment and lipogenesis. Short-term DEN treatment is characterized by a highly increased monocyte/macrophage ratio [20]. While both genotypes exhibited such an increased monocyte/macrophage ratio as assessed by flow cytometric analysis, the ratio was significantly lower in ls*Ttp*-KO mice compared to wild-types (Figure 1D). No difference could be observed between the genotypes in the sham-treated group (Figure 1D). In the long-term model, there is only a very mild inflammation, which was somewhat lower in ls*Ttp*-KO mice.

**Figure 2:**
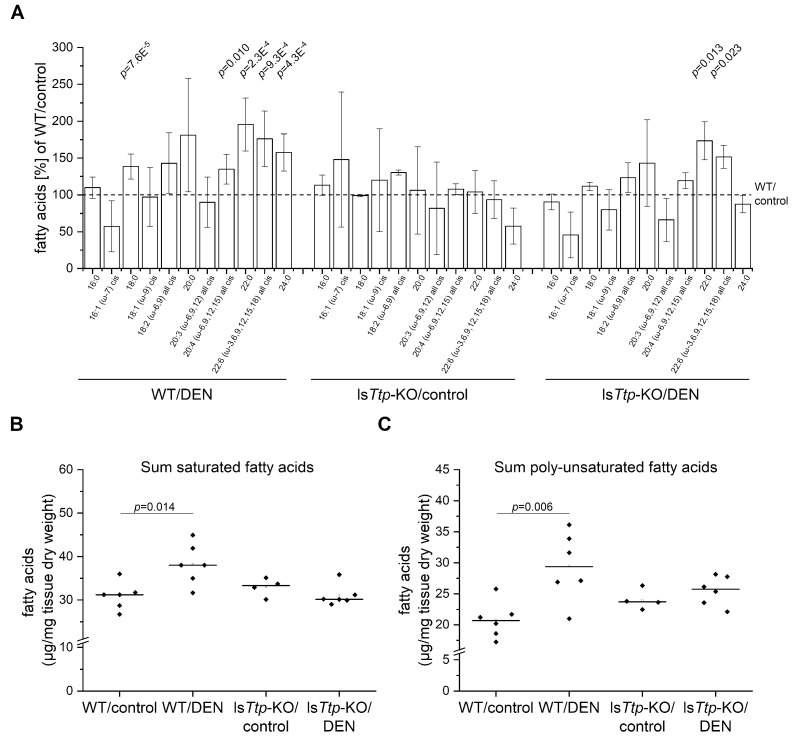
Hepatic fatty acid (FA) profile in short-term DEN-treated ls*Ttp*-KO mice. (**A**): Overview of hepatic FAs in the four comparison groups. The values for the represented FAs are normalized to the corresponding FA in the WT/control, which is set to 100% and is illustrated by the dashed line. Statistical differences refer to the respective control, which means that WT/DEN is compared with the WT/control, and ls*Ttp*-KO/DEN is compared with the ls*Ttp*-KO/control. Error bars indicate SD. Graphs for single FAs are shown in supplementary Figure S1. (**B**): Sum-saturated FAs (14:0, 16:0, 17:0, 18:0, 20:0, 22:0, 24:0). (**C**): Sum-polyunsaturated FAs (18:2 (ω-6,9) all cis, 20:2 (ω-6,9) all cis, 20:3 (ω-6,9,12) all cis, 20:4 (ω-6,9,12,15) all cis, 22:5 (ω-3,6,9,12,15) all cis, 22:6 (ω-3,6,9,12,15,18) all cis). *n* = 6 (WT/control), 6 (WT/DEN), 4 (ls*Ttp*-KO/control), 6 (ls*Ttp*-KO/DEN). Rhombi illustrate single data points, horizontal black lines illustrate median, and white rectangles illustrate means. Significant *p* values (α < 0.05) are shown. (14:0 = myristic acid, 16:0 = palmitic acid, 16:1 (ω-7) cis = palmitoleic acid, 17:0 = margaric acid, 18:0 = stearic acid, 18:1 (ω-9) cis = oleic acid, 18:2 (ω-6,9) all cis = linoleic acid, 20:0 = arachidic acid, 20:2 (ω-6,9) all cis = eicosadienoic acid, 20:3 (ω-6,9,12) all cis = eicosatrienoic acid, 20:4 (ω-6,9,12,15) all cis = arachidonic acid, 22:0 = behenic acid, 22:5 (ω-3,6,9,12,15) all cis = docasapentaenoic acid, 22:6 (ω-3,6,9,12,15,18) all cis = docosahexaenoic acid, 24:0 = lignoceric acid).

The amount and pattern of hepatic lipids are altered during inflammatory conditions and contribute to hepatocarcinogenesis [18,21,22]. Accordingly, short-term DEN treatment also induces lipogenesis [18]. Fatty acid profiling revealed a significant increase in the sum of both saturated fatty acids and poly-unsaturated fatty acids in DEN-treated wild-type livers (Figure 2A–C). However, this increase was less pronounced in ls*Ttp*-KO livers (Figure 2A–C). Profiling of individual fatty acids revealed an increase of individual, but not all, fatty acids, which was almost abrogated in the DEN-treated ls*Ttp*-KO mice (Figure 2A, supplementary Figure S1).

**Figure 3:**
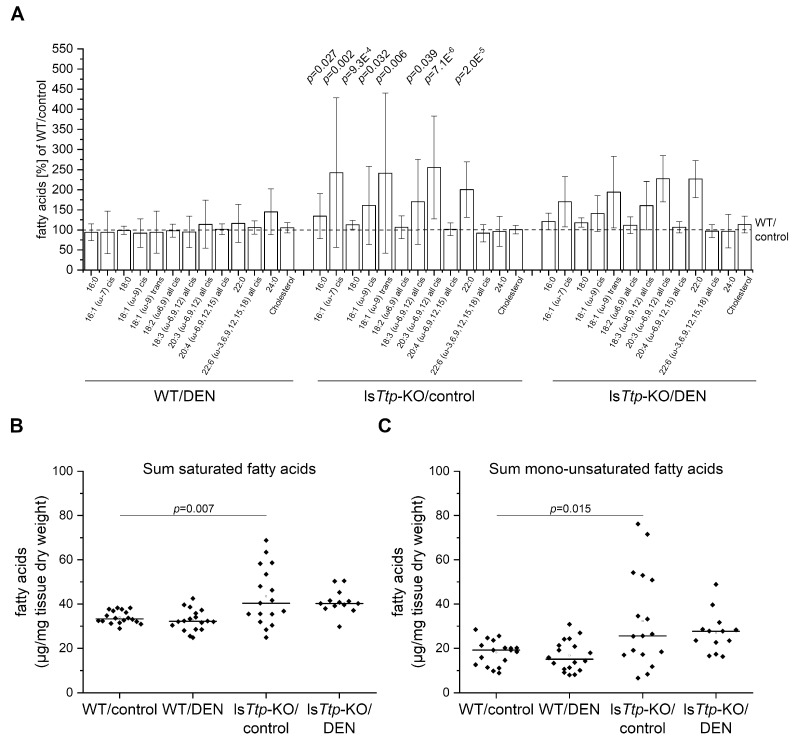
Hepatic fatty acid (FA) profile in long-term DEN-treated ls*Ttp*-KO mice. (**A**): Overview of hepatic FAs in the four comparison groups. The values for the represented FAs are normalized to the corresponding FA in the WT/control, which is set to 100% and is illustrated by the dashed line. Statistical differences refer to the respective partner undergoing the same treatment but different genotype, which means that the WT/control is compared with the ls*Ttp*-KO/control and WT/DEN is compared with ls*Ttp*-KO/DEN. Error bars indicate SD. Graphs for single FAs are shown in supplementary Figure S2. (**B**): Sum saturated FAs (14:0, 16:0, 17:0, 18:0, 20:0, 22:0, 24:0). (**C**): Sum-polyunsaturated FAs (18:2 (ω-6,9) all cis, 20:2 (ω-6,9) all cis, 20:3 (ω-6,9,12) all cis, 20:4 (ω-6,9,12,15) all cis, 22:5 (ω-3,6,9,12,15) all cis, 22:6 (ω-3,6,9,12,15,18) all cis). (**B**)+(**C**): *n* = 6 (WT/control), 6 (WT/DEN), 4 (ls*Ttp*-KO/control), 6 (ls*Ttp*-KO/DEN). Rhombi illustrate single data points, horizontal black lines illustrate median, and white rectangles illustrate means. Significant *p* values (α < 0.05) are shown. (14:0 = myristic acid, 16:0 = palmitic acid, 16:1 (ω-7) cis = palmitoleic acid, 17:0 = margaric acid, 18:0 = stearic acid, 18:1 (ω-9) cis = oleic acid, 18:2 (ω-6,9) all cis = linoleic acid, 20:0 = arachidic acid, 20:2 (ω-6,9) all cis = eicosadienoic acid, 20:3 (ω-6,9,12) all cis = eicosatrienoic acid, 20:4 (ω-6,9,12,15) all cis = arachidonic acid, 22:0 = behenic acid, 22:5 (ω-3,6,9,12,15) all cis = docasapentaenoic acid, 22:6 (ω-3,6,9,12,15,18) all cis = docosahexaenoic acid, 24:0 = lignoceric acid).

Since reprogramming of energy metabolism has been described as a hallmark of cancer [6], we also assessed fatty acid profiles in livers after long-term (six months) DEN treatment. However, no significant difference in the amount of fatty acids upon DEN treatment was observed in either genotype (Figure 3A–C, supplementary Figure S2). Interestingly, though, sham-treated ls*Ttp*-KO mice showed distinctly increased levels of saturated and mono-unsaturated fatty acids compared to wild-types (Figure 3A–C, supplementary Figure S2), suggesting that ls*Ttp*-KO mice change their phenotype over time.

### 2.3. Effects of TTP on Hallmarks of Cancer

**Figure 4:**
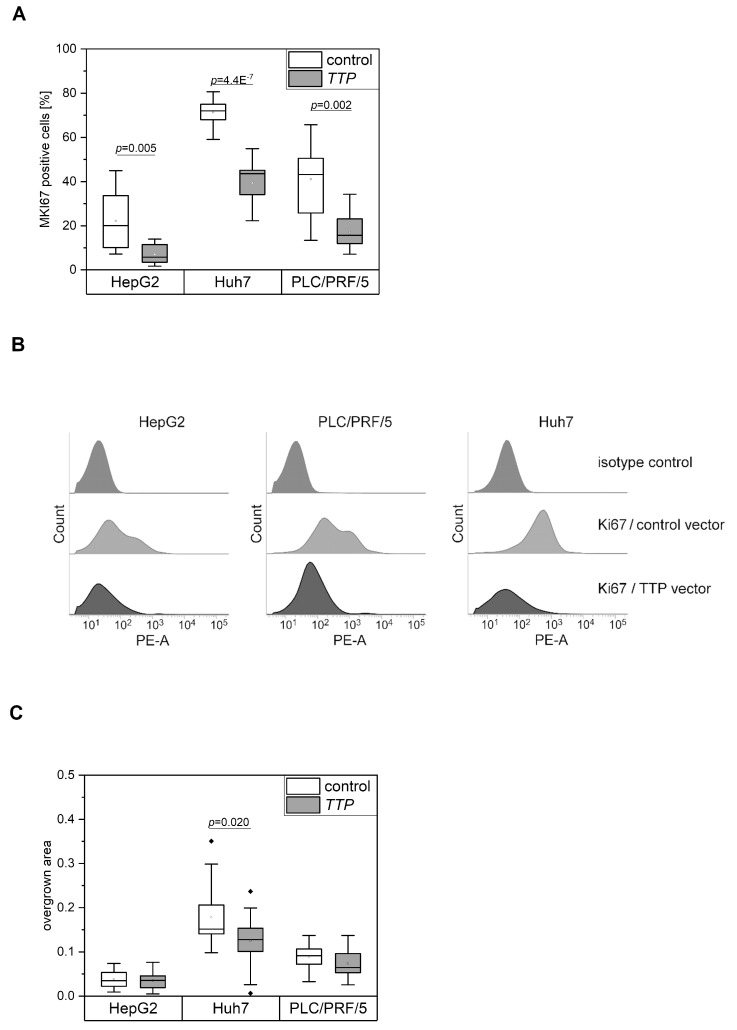
Proliferation and migration of TTP-overexpressing hepatoma cells. (**A**): Proliferation of cells transfected with either *TTP* (gene name *ZFP36*) or a control vector. (**B**): Flow cytometric analysis of the proliferation marker MKI67 in TTP-overexpressing (*TTP* vector) and control cells (control vector). The isotype controls represent the control cells. Representative histograms of MKI67 flow cytometric analyses are shown. *n* = 3; triplicates. (**C**): Migration of HepG2, Huh7, and PLC/PRF/5 cells transfected with either a *TTP* or a control vector. The difference between the open image area t(0) and t(24) was considered as an overgrown area. n = 5–6; quadruplicates. Statistical difference: *: *p* ≤ 0.05; **: *p* ≤ 0.01; ***: *p* ≤ 0.001.

Our data suggested tumor-promoting actions of TTP by supporting tumor initiation. In order to clarify the role of TTP during tumor progression, TTP expression was investigated with respect to several hallmarks of cancer, among which sustaining proliferation might be the most important one. We therefore aimed to investigate a potential action of TTP on cell proliferation by MKI67 staining and flow cytometry in stably overexpressing cell lines. However, cells stably transfected with the overexpressing plasmid did not grow at all. Thus, the proliferation ability of transiently TTP-overexpressing cells was investigated. The proliferation in three different human hepatoma cell lines, i.e., HepG2, PLC/PRF/5, and Huh7 cells was dramatically decreased after TTP overexpression (Figure 4A,B), rather suggesting tumor-suppressing actions of TTP. In line with these findings, we observed that baseline expression of TTP was almost absent in all three cancer cell lines.

Migration as another hallmark of cancer represents a prerequisite of tumor cells to metastasize [6]. We determined the migratory potential of the cells by a scratch assay in TTP-overexpressing or vector control cells. The migratory ability of PLC/PRF/5 and HepG2 cells, but not of Huh7 cells, was inhibited by TTP (Figure 4C), further supporting the tumor-suppressing actions of TTP.

**Figure 5:**
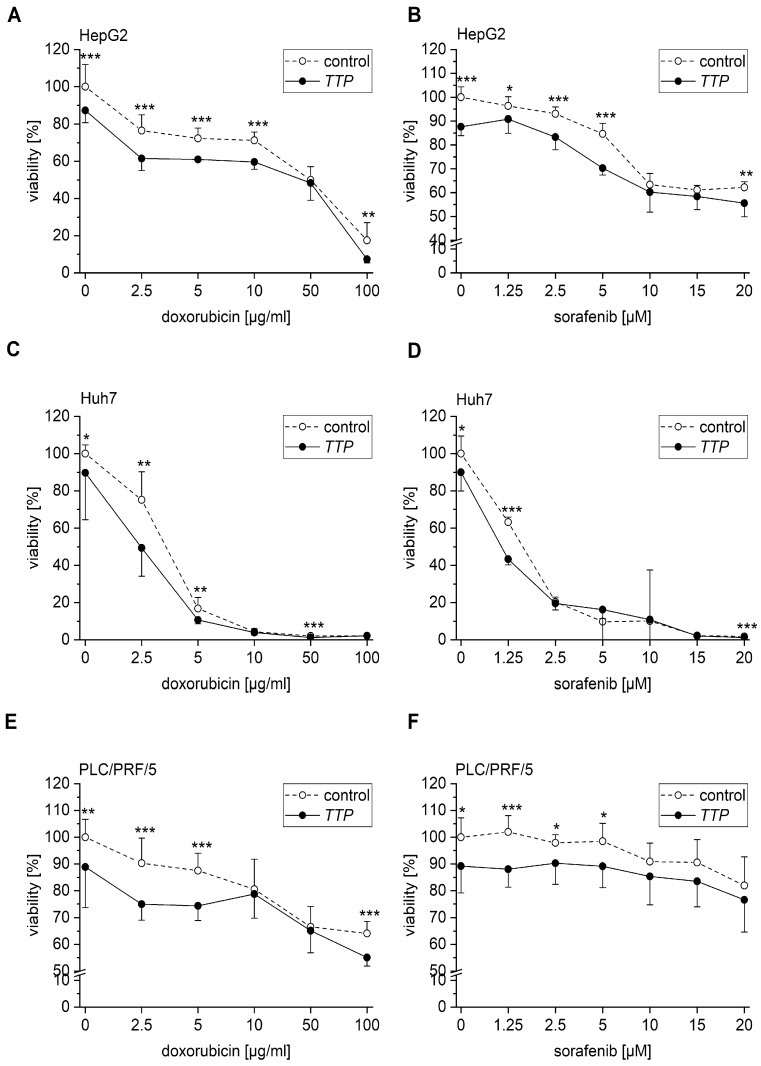
Effects of TTP overexpression on chemoresistance in hepatoma cells. Cells were transfected with either TTP (gene name ZFP36) or a control vector. 24 h after transfection, cells were treated with different concentrations of doxorubicin or sorafenib. Cell viability was determined via 3-(4,5-dimethylthiazol-2-yl)-2,5-diphenyltetrazolium bromide (MTT) assay. (**A**,**B**): HepG2 cells treated with doxorubicin (**A**) or sorafenib (**B**). (**C**,**D**): Huh7 cells treated with doxorubicin (**C**) or sorafenib (**D**). (**E**,**F**): PLC/PRF/5 cells treated with doxorubicin (**E**) or sorafenib (**F**). *n* = 3 (for untreated controls *n* = 6); quadruplicates. Statistical difference: *: *p* ≤ 0.05; **: *p* ≤ 0.01; ***: *p* ≤ 0.001.

As a parameter of chemosensitivity, TTP-overexpressing cells, as well as control HepG2, PLC/PRF/5, and Huh7 cells, were treated with either sorafenib or doxorubicin. The results suggested an impact of TTP overexpression on chemosensitivity in all three cell lines (Figure 5A–F). However, the viability of untreated TTP-overexpressing cells was significantly lower than the number of untreated control cells in all three cell lines (Figure 5A–F). Therefore, the evaluation was adjusted in a way that TTP-overexpressing and control cells were normalized to the control cells. This revealed a less dramatically decreased, but still significantly different chemosensitivity (Supplementary Figure S3).

### 2.4. Expression Changes of Potential TTP Targets

**Figure 6:**
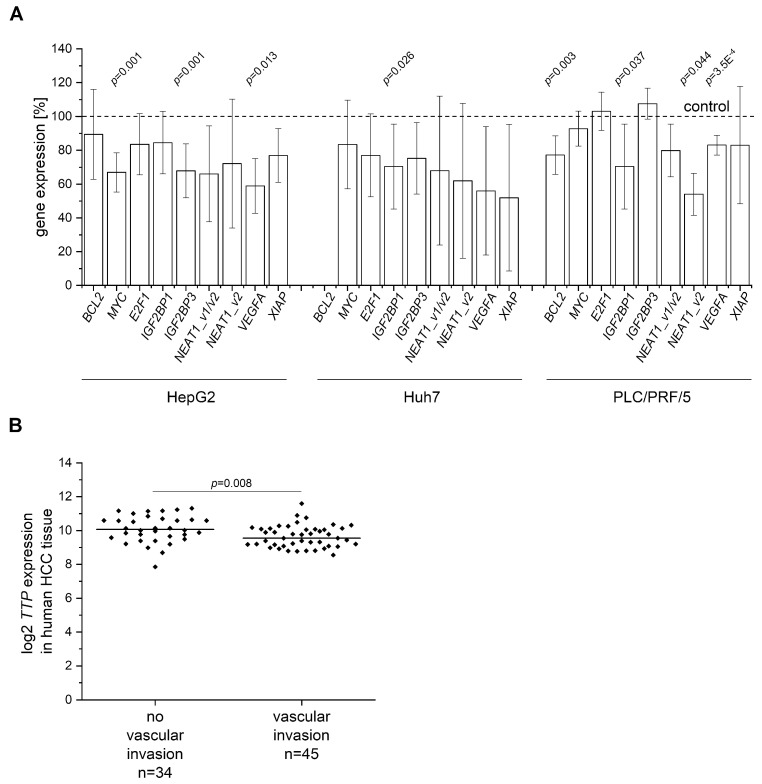
Oncogene expression in TTP-overexpressing hepatoma cells. (**A**) Expression levels normalized to cells treated with a control vector were determined in HepG2, Huh7, and PLC/PRF/5 cells by qPCR. *BCL2* was not determined in Huh7 cells since mRNA expression was below the detection limit. *n* = 2; triplicates. (**B**): *TTP* (gene name *ZFP36*) expression in human hepatocellular carcinoma (HCC) grouped into tumors positive (*n* = 45) or negative (*n* = 34) regarding vascular invasion (GSE20238). Statistical difference: *: *p* ≤ 0.05; **: *p* ≤ 0.01; ***: *p* ≤ 0.001. (*BCL2*: B-cell lymphoma 2, *MYC*: c-Myc, *E2F1*: transcription factor E2F1, *IGF2BP1*: insulin-like growth factor 2 mRNA-binding protein 1, *IGF2BP3*: insulin-like growth factor 2 mRNA-binding protein 3, *NEAT1_v1/v2*: nuclear enriched abundant transcript 1, *NEAT1_v2*: long transcript variant of nuclear enriched abundant transcript 1, *VEGFA*: vascular endothelial growth factor A, *XIAP*: X-linked inhibitor of apoptosis protein).

Since TTP represents an mRNA destabilizing factor, we hypothesized that TTP’s tumor-suppressing actions were caused by an altered expression of its target genes, i.e., that TTP overexpression resulted in a downregulation of oncogenes, some of which are able to induce angiogenesis as a hallmark of cancer [6]. Therefore, the expression of the oncogenes B-cell lymphoma 2 (*BCL2*), c-Myc (*MYC*), transcription factor E2F1 (*E2F1*), vascular endothelial growth factor A (*VEGFA*), and X-linked inhibitor of apoptosis protein (*XIAP*), which have been shown to be TTP targets in non-liver tissues [23,24], was checked. To confirm the validity of these targets, we analyzed the sequences of their 3’-UTRs and found TTP binding-motifs [25] in abundance (Supplementary Material 1). Interestingly, AREs were also predicted in several yet unknown TTP targets, which had been suggested as tumor promoting genes and were therefore also analyzed in TTP-overexpressing cells. One of them is the long transcript variant of nuclear enriched abundant transcript 1 (*NEAT1_v1/v2*), *NEAT1_v2* (Supplementary Material). Two other ARE containing genes represent RBPs themselves: the insulin-like growth factor 2 mRNA-binding protein 1 (*IGF2BP1*) and the insulin-like growth factor 2 mRNA-binding protein 3 (*IGF2BP3*) (Supplementary Material), which both promote hepatic tumor progression [8,26]. In HepG2 and Huh7 cells, all analyzed genes tended to be less expressed after TTP overexpression (Figure 6A). *MYC*, *IGF2BP3*, and *VEGFA* were significantly lowered in TTP-overexpressing HepG2 cells (Figure 6A). In TTP-overexpressing Huh7 cells, *IGF2BP1* was the only gene that was significantly decreased (Figure 6A). *BCL2*, *IGF2BP1*, *NEAT1_v2*, and *VEGFA* were significantly lowered in PLC/PRF/5 cells (Figure 6A). Since *VEGFA*, a promoter of invasion in HCC [27], was less expressed in HepG2 and PLC/PRF/5 cells, we hypothesized that TTP might play a role in HCC vascular invasion. Therefore, *TTP* (gene name *ZFP36*) expression was evaluated in gene expression data from human HCC samples showing vascular invasion compared to samples without vascular invasion. In fact, *TTP* expression was slightly but significantly decreased in cancer tissues showing vascular invasion (Figure 6B).

### 2.5. TTP (gene name ZFP36) Expression in Human HCC Tissue

**Figure 7:**
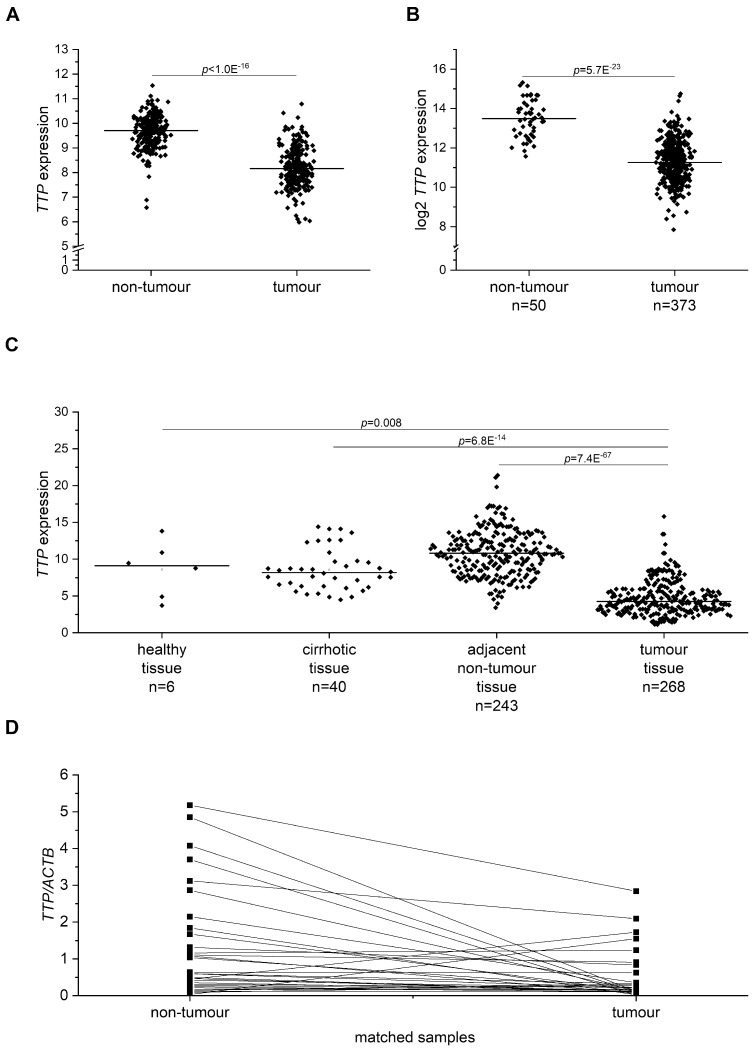
TTP (gene name *ZFP36*) mRNA expression in human tumor and non-tumor liver tissue. (**A**): TTP mRNA expression in 247 human HCC samples relative to the mean of 239 non-tumor liver tissue samples (µ normal) (GSE14520). (**B**): TTP mRNA expression in tumor (*n* = 373) and non-tumor (*n* = 50) tissue (The Cancer Genome Atlas (TCGA)). (**C**): TTP mRNA expression in healthy, cirrhotic, adjacent non-tumor, and tumor liver tissues (500 samples; GSE25097). (**D**): TTP mRNA levels isolated of tumor and matched adjacent non-tumor tissues (*n* = 31).

Since TTP (gene name *ZFP36*) has been shown to be downregulated in different human cancer types, including HCC [14], an extensive expression analysis of TTP mRNA in publicly available HCC data sets was performed: In a microarray data set comprising almost 250 human hepatitis B virus(HBV)-derived HCC samples, in The Cancer Genome Atlas (TCGA) data comparing 373 HCC with 50 non-tumor liver tissue samples, and in a data set comparing HCC tissue with healthy, cirrhotic, and non-tumor tissue of HCC patients. In all data sets, TTP mRNA levels were significantly lower in HCC than in non-tumor tissue (Figure 7A–C). No differential expression was observed for cirrhotic tissue, but the number of normal liver tissue samples was very low (Figure 7C). qPCR analysis of TTP mRNA expression in a set of human liver tumor samples from mixed etiologies and matched normal samples confirmed downregulation of TTP in tumor tissue (Figure 7D).

## 3. Discussion

TTP repression has been described in different human cancers [13,14], and a loss of functional TTP can modulate diverse tumorigenic phenotypes [15]. In this study, we were able to confirm a downregulation of TTP (gene name *ZFP36*) in HCC tissues and tumor suppressor functions of TTP in a set of hallmarks of cancer employing three different hepatoma cell lines [13]. 

Employing an in vivo hepatocarcinogenesis model, our findings are the first to report a role of TTP in carcinogenesis. Since HCC develops based on chronic inflammation and metabolic alterations, a lower number of tumors in ls*Ttp*-KO animals might be connected to the recently described promotion of metabolic liver disease by TTP [16]. 

Although total ls*Ttp*-KO mice show a severe inflammatory phenotype [28] and myeloid-specific ls*Ttp*-KO mice are highly susceptible to lipopolysaccharide treatment [29], a hepatocyte-specific knockout of TTP seemed to have a rather inhibitory effect on inflammation as observed in the short-term mouse experiment: the monocyte to macrophage ratio of DEN-treated knockout mice was decreased compared to the DEN-treated wild type mice. This increase in the monocyte to macrophage ratio has been reported before in DEN-treated animals [20]. 

Although TTP knockdown has been shown to induce monocyte infiltration into three-dimensional tumor spheroids and macrophage infiltration into murine breast cancer xenografts [30], our data rather suggest that hepatocytic TTP promotes tumorigenesis by driving monocyte infiltration and thus tumor-promoting inflammation. This is further strengthened by TTP-dependent alterations in the hepatic fatty acid profile, which might promote tumorigenesis. During hepatocarcinogenesis in obesity-associated chronic liver disease, lipid accumulation can promote inflammation and vice versa [31]. Feeding ls*Ttp*-KO mice a high-fat diet to model steatohepatitis was described to improve insulin resistance [16]. Thus, hepatocytic TTP, which accounts for more than 95% of the hepatic TTP levels ([16] and supplementary Figure S4), seems to rather facilitate metabolic liver disease. For tumor progression, however, lipid alterations in normal adjacent tissue might be less important than elevated lipids in tumor tissue and tumor stroma [32].

In contrast to tumor initiation, our study suggests an inhibitory role of TTP in a set of hallmarks of cancer as characteristics of tumor progression. In fact, other factors promoting lipid deposition and inflammation can be beneficial for cancer progression. One example represents the fatty acid elongase ELOVL6, which contributes to the progression of pre-tumorous conditions, such as steatosis and steatohepatitis [33]. However, it is downregulated in human liver cancer and its downregulation represents a negative clinical predictor [17,34].

So far, downregulation of TTP in HCC has only been shown in one study using a very small patient cohort with *n* = 24 samples [35]. We herewith were able to confirm the results from the latter study in several large HCC patient cohorts.

It is well known that cell migration is a critical factor for cancer metastasis [36], which can occur already in the early stages of tumor progression [37,38]. TTP has been shown to inhibit the migration ability in prostate cancer, ovarian cancer, gastric cancer, and head and neck squamous cell carcinoma cells [24,39,40,41]. Additionally, TTP was suggested to decrease the metastatic potential in breast cancer [42]. To the best of our knowledge, this study is the first to show a migration- and proliferation-inhibitory effect of TTP in hepatic cells. Others were able to show a TTP-induced decrease of metabolic activity in a methylthiazoletetrazolium assay and adherence in a crystal violet assay [35]. The observed inhibition of proliferation may be the major reason why we failed to establish a stable overexpression of the *TTP*-containing plasmid in HepG2, Huh7, and PLC/PRF/5 cells.

TTP has been shown to downregulate several well-established markers for tumor progression like *BCL2*, *VEGFA*, and *MYC* in non-liver tissue [24,43,44]. In line with these findings, we observed a decreased expression of *MYC*, *VEGFA*, and *BCL2* in liver cancer cells overexpressing TTP. The decreased TTP expression in vascularized HCC tissue further supports the hypothesis that TTP might play a role in angiogenesis, which is a hallmark of tumor progression [6]. 

The different expression levels of the analyzed genes comparing the three cell lines might be explained by the distinct heterogeneity of liver cancer itself [45]. According to this, the three analyzed cell lines also have rather different phenotypes [46,47,48].

Several targets of TTP (e.g., *BCL2*, *VEGFA*, and *MYC*) are associated with a poor chemosensitivity [24]. Chemoresistance is widespread in HCC and characterizes tumor progression [49]. Interestingly, TTP overexpression in PLC/PRF/5 cells decreased the expression of the long transcript variant of the long-non coding RNA *NEAT1*, *NEAT1_v2*, which has been reported to enhance chemoresistance in different cancer cell lines, including hepatoma cells [50,51]. *NEAT1* is one of the least stable long non-coding RNAs [52], a presumed tumor promoter, and associated with chemoresistance [50,53]. TTP has been described to mediate chemosensitivity to cisplatin in head and neck cancer cells [44]. In line with these findings, TTP improved chemoresistance towards sorafenib, an approved drug for systemic liver cancer therapy, as well as towards doxorubicin, which is widely used in chemoembolization [49,54]. A connection between chemoresistance and TTP has also previously been suggested for breast cancer [55]. However, the main effect could be explained by a reduced viability due to TTP overexpression. 

## 4. Materials and Methods

### 4.1. Animals

All animal procedures were performed in accordance with the local animal welfare committee (permission no. 37/2014). Male C57BL/6 mice were kept under controlled conditions regarding temperature, humidity, 12 h day/night rhythm, and food access. C57BL/6 Ttp^fl/fl^ mice carrying flox sites flanking exon 2 of TTP were crossed with albumin-Cre transgenic mice in order to generate liver-specific ls*Ttp*-KO mice [16]. Hepatocyte specific ls*Ttp*-KO was confirmed via qPCR (Supplementary Figure S4). TTP expression was almost absent in ls*Ttp*-KO, suggesting that the predominant TTP expression in the liver is found in hepatocytes. For the long-term experiment, which mimics hepatocarcinogenesis [56,57], wild type and two-week-old male ls*Ttp*-KO mice were intraperitoneally injected with either a 5 mg/kg body weight diethylnitrosamine (DEN) solution or a 0.9% NaCl solution as a sham-control to determine the effects of TTP on hepatic tumor initiation. 22 weeks after the injection, the mice were sacrificed. For the short-term experiment mimicking acute hepatic inflammation, wild type and nine-week-old male ls*Ttp*-KO mice were intraperitoneally injected with either a 100 mg/kg body weight DEN solution or with a 0.9% NaCl solution as a sham-control [9,18,19]. 48 h after the injection, the mice were sacrificed.

### 4.2. Histology

For histological analysis, paraffin-embedded liver tissue specimens were cut into 5 µm sections and stained with hematoxylin and eosin (HE) [9,56,58]. Based on histological analysis, macroscopic tumors were confirmed as tumors.

### 4.3. Determination of Hepatic Fatty Acid Profile in Mice

The fatty acid profile was measured by gas chromatography-mass spectrometry (GC-MS) as previously described [18,59,60,61]. In short, snap-frozen liver tissue samples were pestled in liquid nitrogen and freeze-dried overnight. Aliquots of two to five milligrams of tissue dry weight were hydrolyzed using the fatty acid methyl ester method (FAME) according to Bode et. al. [62]. GC-MS was carried out on an Agilent 6890N gas chromatograph (Agilent Technologies, Waldbronn, Germany) equipped with a 7683B split/splitless injector with autosampler (Agilent Technologies) and coupled to a 5973 electron impact mass selective detector (Agilent Technologies) as previously described [18]. Absolute amounts of FAs were quantified by integration of the peaks in relation to the integral of methyl-nonadecanoate (74208, Merck, Taufkirchen, Germany) as an internal standard and to liver tissue dry weight.

### 4.4. Cell Culture

HepG2, PLC/PRF/5, and Huh7 cells were cultured in RPMI-1640 medium with 10% fetal calf serum, 1% penicillin/streptomycin, and 1% glutamine (Sigma–Aldrich, Taufkirchen, Germany) at 37 °C and 5% CO_2_.

### 4.5. Transient TTP Overexpression

For overexpression experiments, a vector (pZeoSV2(–)) containing the human TTP coding sequence tagged with the human influenza hemagglutinin tag or the vector with the antisense sequence as a control (Ref. No.: V855-01, Invitrogen, Carlsbad, California, USA) was used [63]. The vectors were kindly provided by Prof. Dr. Hartmut Kleinert [64], and the sequence was verified by sequencing. Transient TTP overexpression in hepatoma cells was established by transfection with the vector using jetPEI^TM^ Hepatocyte reagent (102-05N, Polyplus transfection, Illkirch, France) as recommended by the manufacturer. Successful TTP overexpression was confirmed via Western Blot for each experiment.

### 4.6. Cytotoxicity Assay (MTT Assay)

Hepatoma cells were seeded into 96-well plates, transfected with TTP or a control vector, and treated with different concentrations of doxorubicin (Sigma–Aldrich) or sorafenib (Biomol GmbH, Hamburg, Germany) and the respective solvent control. 24 h after the treatment, the cytostatic substances were removed and 5 mg/mL MTT (3-[4,5-dimethylthiazol-2-yl]-2,5- diphenyltetrazolium bromide; thiazolyl blue) (Sigma–Aldrich, Taufkirchen, Germany) in medium were added. After 2 h incubation, the formazan crystals were solved in dimethyl sulfoxide, and the absorbance was measured at 550 nm with 690 nm as reference wavelength in a microplate reader (Tecan Sunrise™, Tecan Group Ltd., Männedorf, Switzerland).

### 4.7. Scratch Assay

Cells were seeded into 12-well plates, transfected with *TTP* or a control vector, and scratched 48 h after transfection with a pipet tip. The first image was taken immediately after the scratch; the second image was taken 24 h after the scratch using a 5× objective. Images were obtained and analyzed with an Axio Observer Z1 epifluorescence microscope equipped with an AxioCam Mrm (Zeiss, Oberkochen, Germany) using a 5× objective. Data were analyzed with the TScratch software (CSElab, Zürich, Switzerland).

### 4.8. Human HCC

Paraffin-embedded liver samples (*n* = 31) from randomly selected pseudonymized HCC patients who underwent liver resection at the Saarland University Medical Center between 2005 and 2010 were obtained as described previously [65]. The study protocol was approved by the local Ethics Committee (47/07). Samples had a mixed etiology, including non-alcoholic steatohepatitis (NASH), alcoholic liver disease, viral hepatitis, hemochromatosis, porphyria, and cryptogenic cirrhosis [65].

For differential *ZFP36* expression between tumor (*n* =247) and non-tumor (*n* = 239) samples, the log2 of an RMA-normalized data set (GSE14520 [66]) of an AffymetrixGeneChip HG-U133A 2.0 was analyzed. Similarly, differential gene expression was analyzed in data set GSE25097 [67] between healthy (*n* = 6), cirrhotic (*n* = 40), adjacent non-tumor (*n* = 243), and tumor tissue (*n* = 268), and in data set GSE20238 [68] between vascular invasive (*n* = 45) and non-invasive (*n* = 34) HCC samples. Differential expression analysis was based on the Kolmogorov–Smirnov test. Pearson correlation was applied to detect correlations between genes of interest.

RNAseq expression data from The Cancer Genome Atlas (TCGA) pan cancer dataset, comparing *ZFP36* expression in tumor and non-tumor liver tissue, was produced via Toil [69]. RSEM [70] reported transcripts per million values were downloaded via the UCSC Xena Browser (https://xenabrowser.net) and comprised 373 primary solid tumor as well as 50 matched non-tumor tissue samples.

### 4.9. qPCR

RNA isolation and reverse transcription were performed as previously described [71]. All samples were estimated in triplicate. Primers and conditions are listed in Supplementary Table S1. The absolute gene expression was normalized to *ACTB* (actin beta, for human samples) or *Ppia/Cyclophilin* (peptidylprolyl isomerase A, for murine samples) mRNA values. Stability of the housekeeping genes was tested according to geNorm [72] and NormFinder [73].

### 4.10. Flow Cytometry

Flow cytometric analysis of murine liver leukocyte composition was performed as described previously [20,74]. To determine the amount of leukocytes, 1 µg of the FITC (fluorescein isothiocyanate) mouse anti-mouse CD45.2 Clone 104 antibody (#561874, BD Biosciences, Heidelberg, Germany) in 100 µL was used. To detect the composition of leukocytes, 0.5 µg of each antibody were added: APC rat anti-mouse Ly6G Clone 1A8 (#560599), APC-R700 rat anti-mouse CD11b Clone M1/70 (#564985), BV421 rat anti-mouse Ly6C Clone AL-21 (#562727), BV510 mouse anti-mouse NK 1.1 Clone PK136 (#563096), PE hamster anti-mouse CD11c Clone HL3 (#55740, all from BD Biosciences), and FITC human anti-mouse F4/80 clone REA126 (#130-102-327 from Miltenyi, Bergisch Gladbach, Germany). To determine the composition of the leukocytes, the following gating strategy was applied: FSClow debris and erythrocytes, and multiplets with a non-linear SSC-A/SSC-H ratio were excluded. Viability was determined by 7-AAD staining. Viable cells (7-AAD^−^) were analyzed for CD11b and Cd11c expression. Myeloid dendritic cells were defined as CD11b^+^ CD11c^hi^ cells, and neutrophils were identified as Ly6G^+^ cells within the CD11b^+^ CD11c^−^ population. CD11b^+^ CD11c^−^ Ly6G^−^ NK1.1^−^ cells were further divided into subpopulations according to their Ly6C and F4/80 expression, i.e. macrophages (Ly6C^lo^ F4/80^hi^) and monocytes (Ly6C^hi^ F4/80^lo^), following Blériot et. al. and Ramachandran et al. [75,76]. All gates were defined by using fluorescence minus one (FMO) controls. Flow cytometric analysis of human hepatoma cell proliferation was performed as described previously [56] using the PE (phycoerythrin) Mouse Anti-Human Ki-67 Set (#5113743, BD Biosciences). Graphical illustrations were performed with FACSuite.

### 4.11. Statistical Analysis

Data analysis and statistics of experimental data were performed using the Origin software (OriginPro 8.6G, 2018b, and 2019; OriginLabs, Northampton, MA, USA). All data are displayed either as columns with mean values ± SD or as individual values and boxplots (± interquartile) range with mean (square) and median (line). Normality was tested with the Shapiro–Wilk algorithm. Grubbs test was performed to identify possible outliers. Depending on normality, one-way ANOVA followed by Bonferroni post-hoc test or Kruskal–Wallis–ANOVA followed by Mann–Whitney test was performed. Two-way ANOVA followed by Scheffe post-hoc test was performed on data belonging to four different comparison groups (Figure 1, Figure 2 and Figure 3 and supplementary Figures S1, S2 and S4). Fisher’s-exact test was used for categorical data. Differences were considered statistically significant with *p* ≤ 0.05.

## 5. Conclusions

In conclusion, our data suggest that hepatocytic TTP plays an inflammation- and lipid-dependent role in promoting hepatic tumor initiation. In liver cancer progression, TTP exerts a major inhibitory effect. The potential limit of translatability of rodent models in HCC research due to differences in anatomy, physiology, and mechanisms of carcinogenesis [76] has to be addressed in future studies.

## Supplementary Materials

The following are available online at https://www.mdpi.com/2072-6694/11/11/1754/s1, Figure S1: Hepatic fatty acids (FAs) in short-term sham- (control) or DEN-treated WT and ls*Ttp*-KO mice, Figure S2: Hepatic fatty acids (FAs) in long-term sham- (control) or DEN-treated WT and ls*Ttp*-KO mice, Figure S3: Effects of TTP overexpression on chemoresistance in hepatoma cells (normalised to control vector), Figure S4: *Zfp36* mRNA levels in ls*Ttp*-KO and WT animals, Table S1: Primer sequences of human and murine genes, Other Supplementary Material: ARE in TTP target genes, Supplementary Material 1: ARE in TTP target genes.
