# Supplementary material for: The mRNA-binding Protein TTP/ZFP36 in Hepatocarcinogenesis and Hepatocellular Carcinoma
Source: Cancers (Basel). 2019 Nov 8;11(11):1754. doi: 10.3390/cancers11111754 (PMC6896064; doi:10.3390/cancers11111754)
Supplement: Supplementary file 1 [file cancers-11-01754-s001.pdf]

The mRNA-binding protein TTP promotes hepatocarcinogenesis but inhibits tumour progression in liver cancer

Tarek Kröhler, Sonja M. Kessler, Kevan Hosseini, Markus List, Ahmad Barghash, Sonika Patial, Stephan Laggai, Katja Gemperlein, Johannes Haybaeck, Rolf Müller, Volkhard Helms, Marcel H. Schulz, Jessica Hoppstädter, Perry J. Blackshear, Alexandra K. Kiemer

Supplementary material

Supplementary Figures:

Figure S1: Hepatic fatty acids (FAs) in short-term sham- (control) or DEN-treated WT and ls*Ttp*-KO mice

Figure S2: Hepatic fatty acids (FAs) in long-term sham- (control) or DEN-treated WT and ls*Ttp*-KO mice

Figure S3: Effects of TTP overexpression on chemoresistance in hepatoma cells (normalised to control vector)

Figure S4: *Zfp36* mRNA levels in ls*Ttp*-KO and WT animals

Supplementary Tables:

Table S1: Primer sequences of human and murine genes

Other Supplementary Material:

ARE in TTP target genes

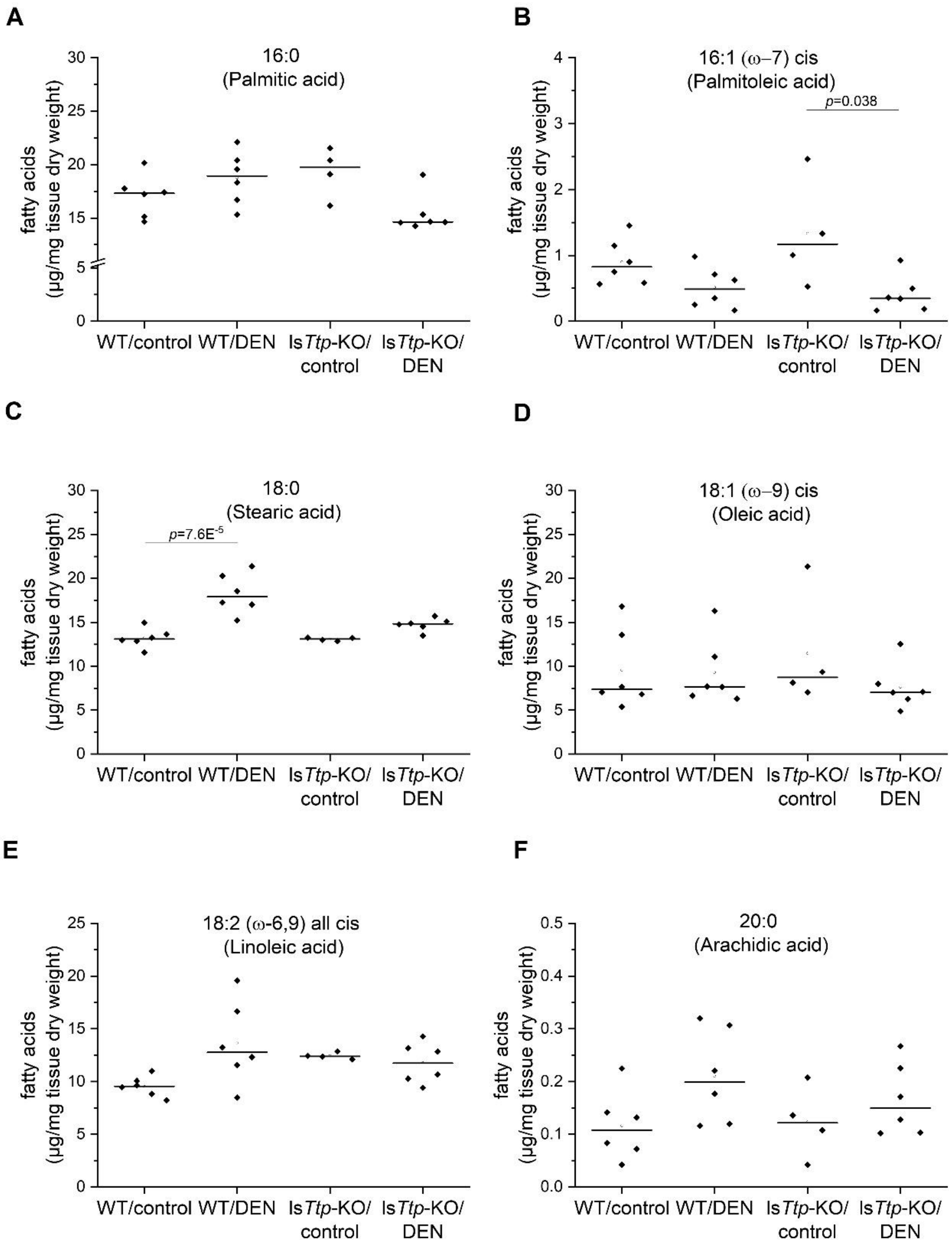
A
16:0
(Palmitic acid)
fatty acids
(µg/mg tissue dry weight)
WT/control WT/DEN IsTtp-KO/control IsTtp-KO/DEN
B
16:1 (ω–7) cis
(Palmitoleic acid)
p=0.038
C
18:0
(Stearic acid)
p=7.6E-5
D
18:1 (ω–9) cis
(Oleic acid)
E
18:2 (ω-6,9) all cis
(Linoleic acid)
F
20:0
(Arachidic acid)

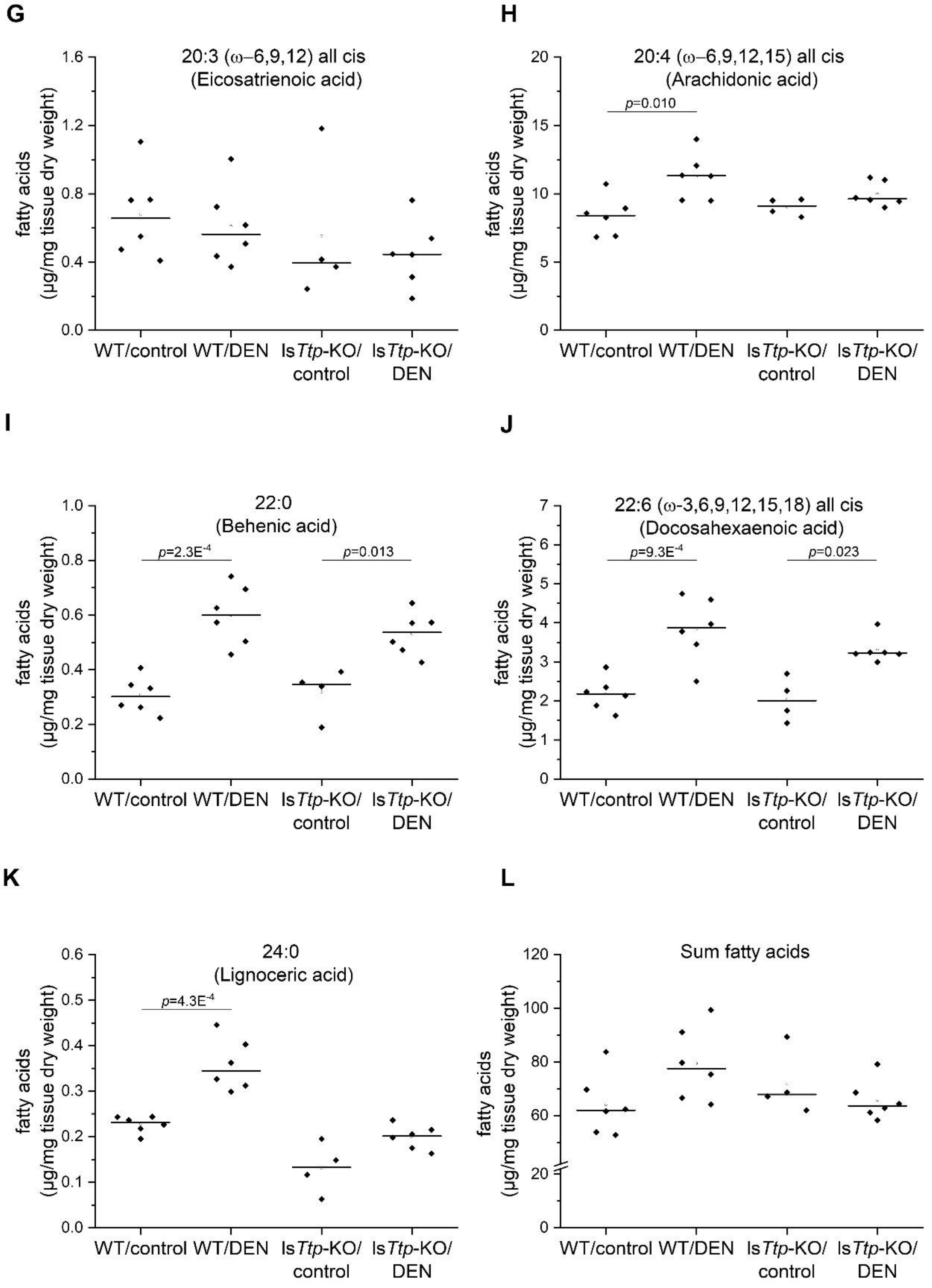

Supplementary Figure S1. Hepatic fatty acids (FAs) in short-term sham- (control) or DEN-treated WT and ls*Ttp*-KO mice. n = 6 (WT/control), 6 (WT/DEN), 4 (ls*Ttp*-KO/control), 6 (ls*Ttp*-KO/DEN). Rhombi illustrate single data points, horizontal black lines illustrate median and white rectangles illustrate means. Significant p values ($\alpha < 0.05$) are shown.

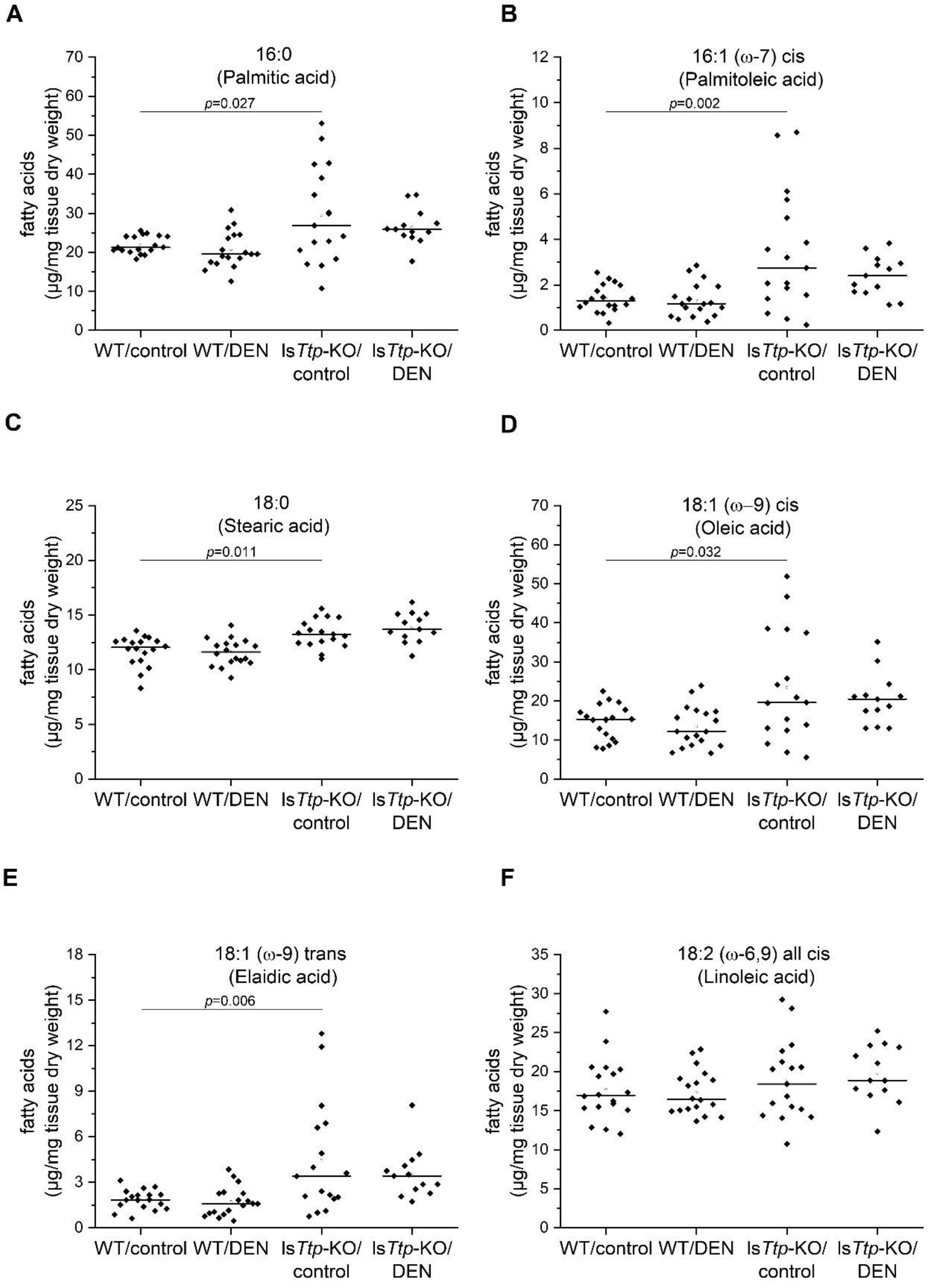
A
16:0
(Palmitic acid)
p=0.027
fatty acids
(µg/mg tissue dry weight)
WT/control
WT/DEN
lsTtp-KO/control
lsTtp-KO/DEN
B
16:1 (ω-7) cis
(Palmitoleic acid)
p=0.002
C
18:0
(Stearic acid)
p=0.011
D
18:1 (ω–9) cis
(Oleic acid)
p=0.032
E
18:1 (ω-9) trans
(Elaidic acid)
p=0.006
F
18:2 (ω-6,9) all cis
(Linoleic acid)

G

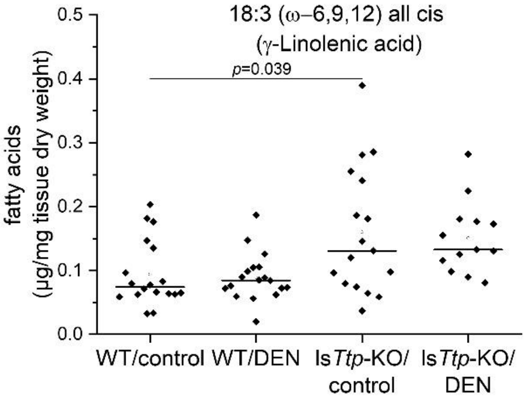

18:3 (ω–6,9,12) all cis
(γ-Linolenic acid)
p=0.039
fatty acids
(µg/mg tissue dry weight)
0.0
0.1
0.2
0.3
0.4
0.5
WT/control
WT/DEN
IsTtp-KO/ control
IsTtp-KO/ DEN

H

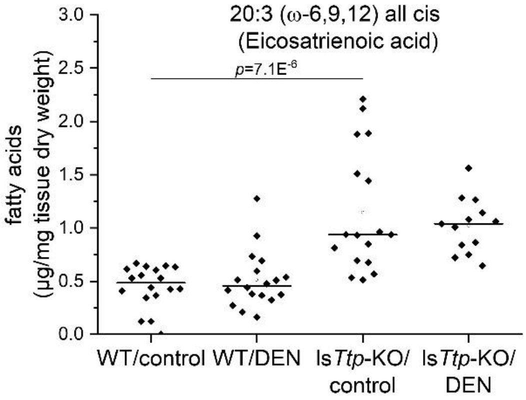

20:3 (ω-6,9,12) all cis
(Eicosatrienoic acid)
p=7.1E-6
fatty acids
(µg/mg tissue dry weight)
0.0
0.5
1.0
1.5
2.0
2.5
3.0
WT/control
WT/DEN
IsTtp-KO/ control
IsTtp-KO/ DEN

I

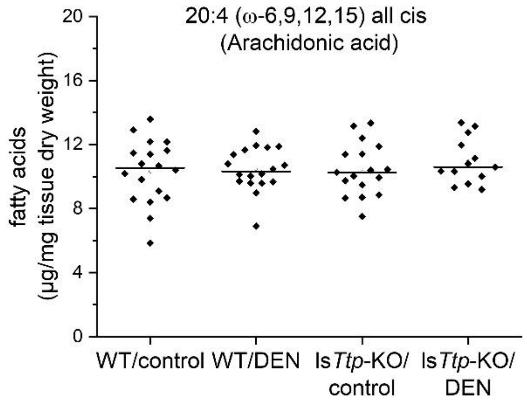

20:4 (ω-6,9,12,15) all cis
(Arachidonic acid)
fatty acids
(µg/mg tissue dry weight)
0
4
8
12
16
20
WT/control
WT/DEN
IsTtp-KO/ control
IsTtp-KO/ DEN

J

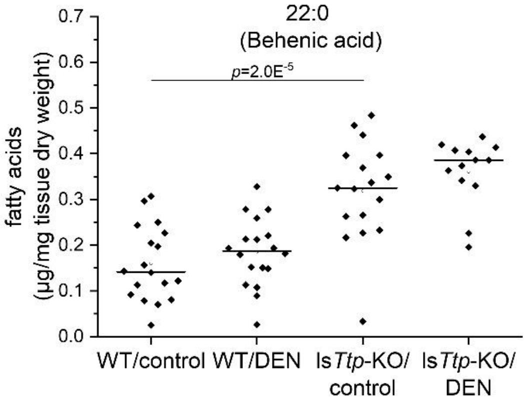

22:0
(Behenic acid)
p=2.0E-5
fatty acids
(µg/mg tissue dry weight)
0.0
0.1
0.2
0.3
0.4
0.5
0.6
0.7
WT/control
WT/DEN
IsTtp-KO/ control
IsTtp-KO/ DEN

K

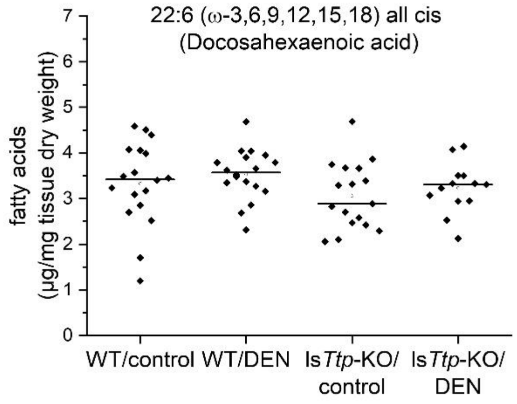

22:6 (ω-3,6,9,12,15,18) all cis
(Docosahexaenoic acid)
fatty acids
(µg/mg tissue dry weight)
0
1
2
3
4
5
6
7
WT/control
WT/DEN
IsTtp-KO/ control
IsTtp-KO/ DEN

L

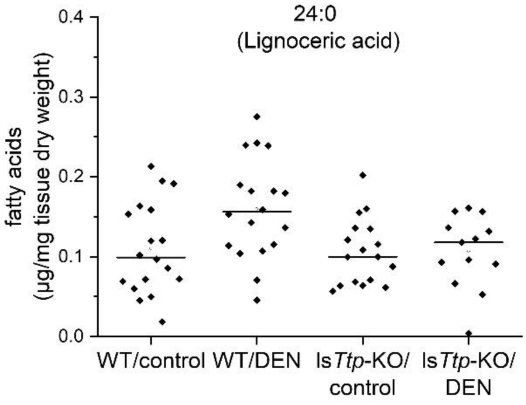

24:0
(Lignoceric acid)
fatty acids
(µg/mg tissue dry weight)
0.0
0.1
0.2
0.3
0.4
WT/control
WT/DEN
IsTtp-KO/ control
IsTtp-KO/ DEN

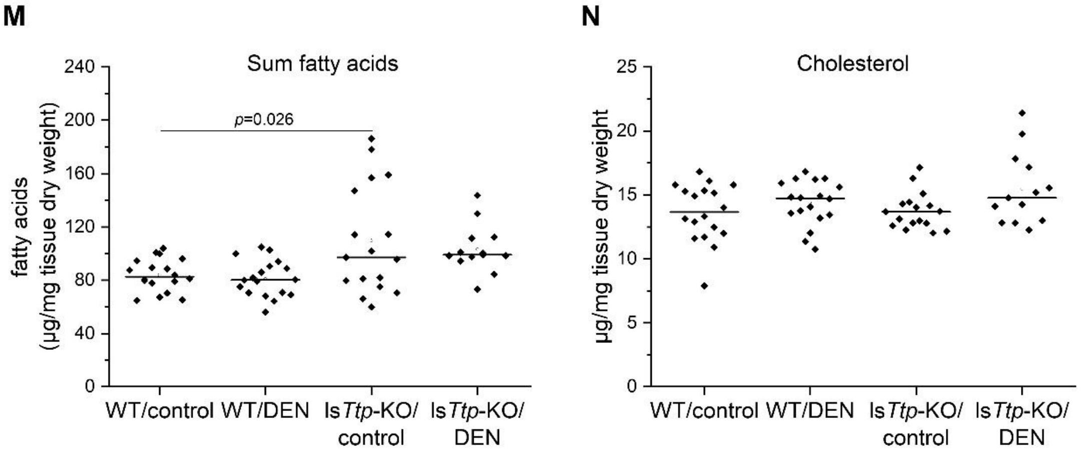

Supplementary Figure S2. Hepatic fatty acids (FAs) in long-term sham- (control) or DEN-treated WT and ls*Ttp*-KO mice. n = 18 (WT/control), 18 (WT/DEN), 17 (ls*Ttp*-KO/control), 13 (ls*Ttp*-KO/DEN). Rhombi illustrate single data points, horizontal black lines illustrate median and white rectangles illustrate means. Significant p values ($\alpha < 0.05$) are shown.

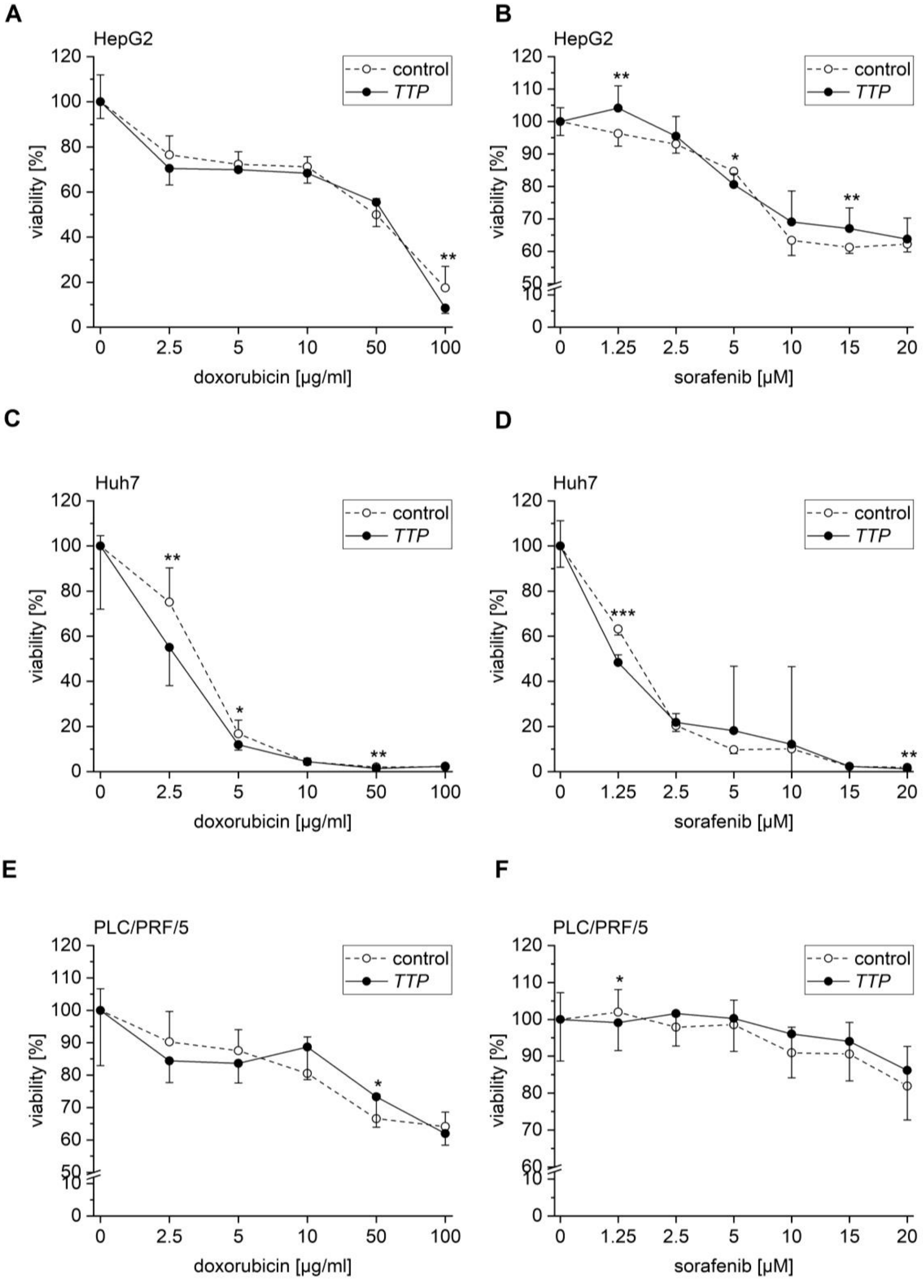

Supplementary Figure S3. Effects of TTP overexpression on chemoresistance in hepatoma cells (normalised to control vector). Cells were transfected with either *TTP* (gene name *ZFP36*) or a control vector. 24 h after transfection, cells were treated with different concentrations of doxorubicin or sorafenib for 24 h. Cell viability was determined *via* MTT assay. Both groups (*TTP* and control vector) are normalised to the viability of the control vector transfected cells without addition of doxorubicin or sorafenib (=100%). (A): HepG2 cells treated with doxorubicin. (B): HepG2 cells treated with sorafenib. (C): Huh7 cells treated with doxorubicin. (D): Huh7 cells treated with sorafenib. (E): PLC/PRF/5 cells treated with doxorubicin. (F): PLC/PRF/5 cells treated with sorafenib. n = 3; quadruplicates. Statistical difference: *: $p \leq 0.05$; **: $p \leq 0.01$; ***: $p \leq 0.001$.

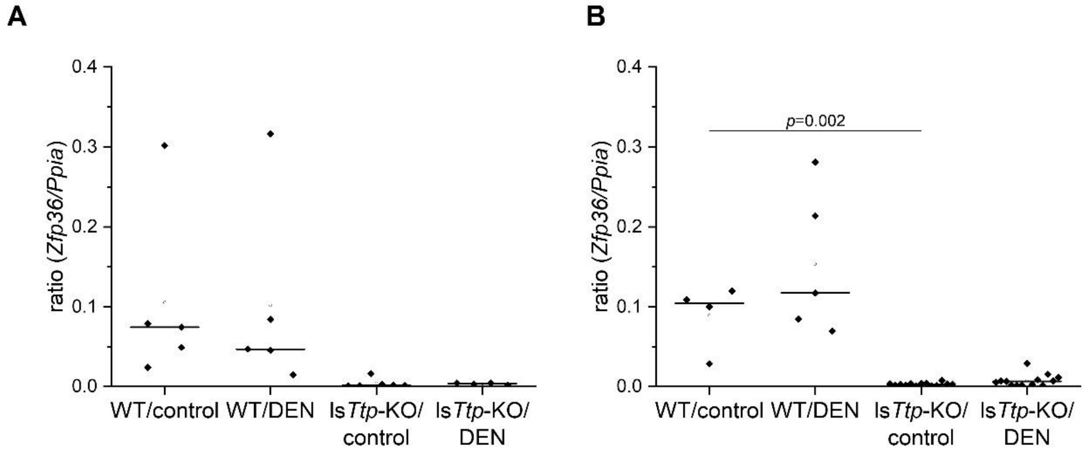

Supplementary Figure S4: *Zfp36* mRNA levels in ls*Ttp*-KO and WT animals. *Zfp36* / *Ppia* mRNA ratio in wild type (WT) and ls*Ttp*-KO (KO) animals injected with either 0.9% NaCl as sham-treatment of DEN following the short-term (A) or long-term (B) protocol.

gene	forward primer sequence 5' → 3'	reverse primer sequence 5' → 3'	gene bank accession no.	AT [°C]	product size [bp]	primer concen-tration [µM]
ACTB	TGCGTGACATTA AGGAGAAG	GTCAGGCAG CTCGTAGCTCT	NM_001101.3	60	107	0.2
BCL2	ACAACATCGCC CTGTGGATGAC	ACTTGTGGCTC AGATAGGCACC	NM_000633.2; NM_000657.2	64	206	0.25
E2F1	AGTTCATCAGC CTTTCCCCACC	CTCCAAGCCCT GTCAGAAATCC	NM_005225.2	59	133	0.25
ELAVL1	GGTGACATCGG GAGAACGAA	CCAAGCTGTGT CCTGCTACT	NM_001419.2	60	142	0.25
IGFBP1	GCCTCCATCAA GATTGCACCAC	AGCTTCACTTCC TCCTTGGGAC	NM_006546.3; NM_001160423.1	62	145	0.2
IGFBP3	TCCCACCCAATT TGTTGGAGCC	GCAGCCCCCGC ATTTTCTTTAC	NM_006547.2	62	113	0.2
MYC	AGCCACAGCAT ACATCCTGTCC	CTCGTCGTTTCC GCAACAAGTC	NM_002467.5; NM_001354870.1	56	79	0.2
NEAT1_ v1/v2	TGCTACAAGGT GGGGAAGACTG	CCCACACCCCA AACAAAACAA	NR_131012.1; NR_028272.1	60	185	0.25
NEAT1_ v2	TTTCAAAGGGA GCAGCAAGGG	ACGGCACAGGC AAATAAGACAC	NR_131012.1	64	199	0.25
ZFP36	TCGCCACCCCA AATACAA	TTCGCTAGGGT TGTGGAT	NM_003407.3	60	99	0.25
VEGFA	CGCTTACTCTCA CCTGCTTCTG	GGTCAACCACT CACACACACAC	NM_001171623.1	60	240	0.25
XIAP	AATAGTGCCAC GCAGTCTACA	CAGATGGCCTG TCTAAGGCAA	NM_001167.3; NM_001204401.1	64	102	0.25
Csnk2a2	GTAAAGGACCC TGTGTCAAAGA	GTCAGGATCTG GTAGAGTTGCT	NM_009974.3	60	85	0.4
Zfp36	CTTCATCCACAA CCCCACC	CAGGGAAGGGC CAGAAAAG	NM_011756.4	59	138	0.25
Ppia	GCGTCTCCTTC GAGCTGTTT	CACCCTGGCAC ATGAATCCT	NM_008907.1	60	138	0.5

Supplementary Table 1: Primer sequences of human and murine genes.

Supplementary Material 1: ARE in TTP target genes.

The following motifs are enriched in TTP targets (Mukherjee et al., 2014): ATTTA (blue), TTTTT (yellow), **CTTTT (fat)**, TTTTC (bordeaux), CTTTC (purple), TATTTATT (green), TTATTTATT (underlined) and TATT (orange).

BCL2 variant alpha (NM_000633.2)

TTTCTGTGAAGCAGAAGTCTGGGAATCGATCTGGAAATCCTCCTAATTTTTACTCCCTCTCCCCGCGACTCCTGA
TTCATTGGGAAGTTTCAAATCAGCTATAACTGGAGAGTGCTGAAGATTGATGGGATCGTTGCCTTATGCATTTGT
TTTGGTTTTACAAAAAGGAAACTTGACAGAGGATCATGCTGTACTTAAAAAATACAACATCACAGAGGAAGTAGA
CTGATATTAACAATACTTACTAATAATAACGTGCCTCATGAAATAAAGATCCGAAAGGAATTGGAATAAAAATTT
CCTGCATCTCATGCCAAGGGGGAAACACCAGAATCAAGTGTTCCGCGTGATTGAAGACACCCCCTCGTCCAAGAA
TGCAAAGCACATCCAATAAAATAGCTGGATTATAACTCCTCTTCTTTCTCTGGGGGCCGTGGGGTGGGAGCTGGG
GCGAGAGGTGCCGTTGGCCCCCGTTG**CTTTT**CCTCTGGGAAGGATGGCGCACGCTGGGAGAACAGGGTACGATAA
CCGGGAGATAGTGATGAAGTACATCCATTATAAGCTGTCGCAGAGGGGCTACGAGTGGGATGCGGGAGATGTGGG
CGCCGCGCCCCCGGGGGCCGCCCCCGCACCGGGCATCTTCTCCTCCCAGCCCGGGCACACGCCCCATCCAGCCGC
ATCCCGGGACCCGGTCGCCAGGACCTCGCCGCTGCAGACCCCGGCTGCCCCCGGCGCCGCCGCGGGGCCTGCGCT
CAGCCCGGTGCCACCTGTGGTCCACCTGACCCTCCGCCAGGCCGGCGACGACTTCTCCCGCCGCTACCGCCGCGA
CTTCGCCGAGATGTCCAGCCAGCTGCACCTGACGCCCTTCACCGCGCGGGGACGCTTTGCCACGGTGGTGGAGGA
GCTCTTCAGGGACGGGGTGAACTGGGGGAGGATTGTGGCCTTCTTTGAGTTCGGTGGGGTCATGTGTGTGGAGAG
CGTCAACCGGGAGATGTCGCCCCTGGTGGACAACATCGCCCTGTGGATGACTGAGTACCTGAACCGGCACCTGCA
CACCTGGATCCAGGATAACGGAGGCTGGGATGCCTTTGTGGAACTGTACGGCCCCAGCATGCGGCCTCTGTTTGA
TTTCTCCTGGCTGTCTCTGAAGACTCTGCTCAGTTTGGCCCTGGTGGGAGCTTGCATCACCCTGGGTGCCTATCT
GGGCCACAAGTGAAGTCAACATGCCTGCCCCAAACAAATATGCAAAAGGTTCACTAAAGCAGTAGAAATAATATG
CATTGTCAGTGATGTACCATGAAACAAAGCTGCAGGCTGTTTAAGAAAAAATAACACACATATAAACATCACACA
CACAGACAGACACACACACACACAACAATTAACAGTCTTCAGGCAAAACGTCGAATCAGCTATTTACTGCCAAAG
GGAAATATCATTTATTTTTTACATTATTAAGAAAAAAAGATTTATTTATTTAAGACAGTCCCATCAAAACTCCTG
TCTTTGGAAATCCGACCACTAATTGCCAAGCACCGCTTCGTGTGGCTCCACCTGGATGTTCTGTGCCTGTAAACA
TAGATTCGCTTTCCATGTTGTTGGCCGGATCACCATCTGAAGAGCAGACGGATGGAAAAAGGACCTGATCATTGG
GGAAGCTGGCTTTCTGGCTGCTGGAGGCTGGGGAGAAGGTGTTCATTCACTTGCATTTCTTTGCCCTGGGGGCTG
TGATATTAACAGAGGGAGGGTTCCTGTGGGGGGAAGTCCATGCCTCCCTGGCCTGAAGAAGAGACTCTTTGCATA
TGACTCACATGATGCATACCTGGTGGGAGGAAAAGAGTTGGGAACTTCAGATGGACCTAGTACCCACTGAGATTT
CCACGCCGAAGGACAGCGATGGGAAAAATGCCCTTAAATCATAGGAAAGTATTTTTTTAAGCTACCAATTGTGCC
GAGAAAAGCATTTTAGCAATTTATACAATATCATCCAGTACCTTAAGCCCTGATTGTGTATATTCATATATTTTG
GATACGCACCCCCCAACTCCCAATACTGGCTCTGTCTGAGTAAGAAACAGAATCCTCTGGAACTTGAGGAAGTGA
ACATTTCGGTGACTTCCGCATCAGGAAGGCTAGAGTTACCCAGAGCATCAGGCCGCCACAAGTGCCTG**CTTTT**AG
GAGACCGAAGTCCGCAGAACCTGCCTGTGTCCCAGCTTGGAGGCCTGGTCCTGGAACTGAGCCGGGGCCCTCACT
GGCCTCCTCCAGGGATGATCAACAGGGCAGTGTGGTCTCCGAATGTCTGGAAGCTGATGGAGCTCAGAATTCCAC
TGTCAAGAAAGAGCAGTAGAGGGGTGTGGCTGGGCCTGTCACCCTGGGGCCCTCCAGGTAGGCCCGTTTTCACGT
GGAGCATGGGAGCCACGACCCTTCTTAAGACATGTATCACTGTAGAGGGAAGGAACAGAGGCCCTGGGCCCTTCC
TATCAGAAGGACATGGTGAAGGCTGGGAACGTGAGGAGAGGCAATGGCCACGGCCCATTTTGGCTGTAGCACATG
GCACGTTGGCTGTGTGGCCTTGGCCCACCTGTGAGTTTAAAGCAAGGCTTTAAATGACTTTGGAGAGGGTCACAA
ATCCTAAAAGAAGCATTGAAGTGAGGTGTCATGGATTAATTGACCCCTGTCTATGGAATTACATGTAAAACATTA
TCTTGTCACTGTAGTTTGGTTTTATTTGAAAACCTGACAAAAAAAAAAGTTCCAGGTGTGGAATATGGGGGTTATC
TGTACATCCTGGGGCATTAAAAAAAAAAATCAATGGTGGGGAACTATAAAGAAGTAACAAAAGAAGTGACATCTTC
AGCAAATAAACTAGGAAATTTTTTTTTCTTCCAGTTTAGAATCAGCCTTGAAACATTGATGGAATAACTCTGTGG
CATTATTGCATTATATACCATTTATCTGTATTAACTTTGGAATGTACTCTGTTCAATGTTTAATGCTGTGGTTGA
TATTTCGAAAGCTGCTTTAAAAAAATACATGCATCTCAGCGTTTTTTTGTTTTTAATTGTATTTAGTTATGGCCT
ATACACTATTTGTGAGCAAAGGTGATCGTTTTCTGTTTGAGATTTTTATCTCTTGATTCTTCAAAAGCATTCTGA
GAAGGTGAGATAAGCCCTGAGTCTCAGCTACCTAAGAAAAACCTGGATGTCACTGGCCACTGAGGAGCTTTGTTT
CAACCAAGTCATGTGCATTTCCACGTCAACAGAATTGTTTATTGTGACAGTTATATCTGTTGTCCCTTTGACCTT
GTTTCTTGAAGGTTTCCTCGTCCCTGGGCAATTCCGCATTTAATTCATGGTATTCAGGATTACATGCATGTTTGG
TTAAACCCATGAGATTCATTCAGTTAAAAATCCAGATGGCAAATGACCAGCAGATTCAAATCTATGGTGGTTTGA
CCTTTAGAGAGTTGCTTTACGTGGCCTGTTTCAACACAGACCCACCCAGAGCCCTCCTGCCCTCCTTCCGCGGGG
GCTTTCTCATGGCTGTCCTTCAGGGTCTTCCTGAAATGCAGTGGTGCTTACGCTCCACCAAGAAAGCAGGAAACC
TGTGGTATGAAGCCAGACCTCCCCGGCGGGCCTCAGGGAACAGAATGATCAGACCTTTGAATGATTCTAATTTTT

AAGCAAAATATTATTTTATGAAAGGTTTACATTGTCAAAGTGATGAATATGGAATATCCAATCCTGTGCTGCTAT
CCTGCCAAAATCATTTTAATGGAGTCAGTTTGCAGTATGCTCCACGTGGTAAGATCCTCCAAGCTGCTTTAGAAG
TAACAATGAAGAACGTGGACGTTTTTAATATAAAGCCTGTTTTGT**CTTTT**GTTGTTGTTCAAACGGGATTCACAG
AGTATTTGAAAAATGTATATATATTAAGAGGTCACGGGGGCTAATTGCTGGCTGGCTGC**CTTTT**GCTGTGGGGTT
TTGTTACCTGGTTTTAATAACAGTAAATGTGCCCAGCCTCTTGGCCCCAGAACTGTACAGTATTGTGGCTGCACT
TGCTCTAAGAGTAGTTGATGTTGCATTTTCCTTATTGTTAAAAACATGTTAGAAGCAATGAATGTATATAAAAGC
CTCAACTAGTCATTTTTTTCTCCTCTT**CTTTT**TTTTCATTATATCTAATTATTTTGCAGTTGGGCAACAGAGAAC
CATCCCTATTTTGTATTGAAGAGGGATTCACATCTGCATCTTAACTGCTCTTTATGAATGAAAAAACAGTCCTCT
GTATGTACTCCTCTTTACACTGGCCAGGGTCAGAGTTAAATAGAGTATATGCACTTTCCAAATTGGGGACAAGGG
CTCTAAAAAAAGCCCCAAAAGGAGAAGAACATCTGAGAACCTCCTCGGCCCTCCCAGTCCCTCGCTGCACAAATA
CTCCGCAAGAGAGGCCAGAATGACAGCTGACAGGGTCTATGGCCATCGGGTCGTCTCCGAAGATTTGGCAGGGGC
AGAAAACTCTGGCAGGCTTAAGATTTGGAATAAAGTCACAGAATTAAGGAAGCACCTCAATTTAGTTCAAACAAG
ACGCCAACATTCTCTCCACAGCTCACTTACCTCTCTGTGTTCAGATGTGGCCTTCCATTTATATGTGATCTTTGT
TTTATTAGTAAATGCTTATCATCTAAAGATGTAGCTCTGGCCCAGTGGGAAAAATTAGGAAGTGATTATAAATCG
AGAGGAGTTATAATAATCAAGATTAAATGTAAATAATCAGGGCAATCCCAACACATGTCTAGCTTTCACCTCCAG
GATCTATTGAGTGAACAGAATTGCAAATAGTCTCTATTTGTAATTGAACTTATCCTAAAACAAATAGTTTATAAA
TGTGAACTTAAACTCTAATTAATTCCAACTGTA**CTTTT**AAGGCAGTGGCTGTTTTTAGACTTTCTTATCACTTAT
AGTTAGTAATGTACACCTACTCTATCAGAGAAAAACAGGAAAGGCTCGAAATACAAGCCATTCTAAGGAAATTAG
GGAGTCAGTTGAAATTCTATTCTGATCTTATTCTGTGGTGT**CTTTT**GCAGCCCAGACAAATGTGGTTACACA**CTT**
TTTAAGAAATACAATTCTACATTGTCAAGCTTATGAAGGTTCCAATCAGATCTTTATTGTTATTCAATTTGGATC
TTTCAGGGATTTTTTTTTTAAATTATTATGGGACAAAGGACATTTGTTGGAGGGGTGGAGGGAGGAAGAATTTT
TAAATGTAAAACATTCCCAAGTTTGGATCAGGGAGTTGGAAGTTTTCAGAATAACCAGAACTAAGGGTATGAAGG
ACCTGTATTGGGGTCGATGTGATGCCTCTGCGAAGAACCTTGTGTGACAAATGAGAAACATTTTGAAGTTTGTGG
TACGACCTTTAGATTCCAGAGACATCAGCATGGCTCAAAGTGCAGCTCCGTTTGGCAGTGCAATGGTATAAATTT
CAAGCTGGATATGTCTAATGGGTATTTAAACAATAAATGTGCAGTTTTAACTAACAGGATATTTAATGACAACCT
TCTGGTTGGTAGGGACATCTGTTTCTAAATGTTTATTATGTACAATACAGAAAAAAATTTTATAAAATTAAGCAA
TGTGAAACTGAATTGGAGAGTGATAATACAAGTCCTTTAGTCTTACCCAGTGAATCATTCTGTTCCATGTCTTTG
GACAACCATGACCTTGGACAATCATGAAATATGCATCTCACTGGATGCAAAGAAAATCAGATGGAGCATGAATGG
TACTGTACCGGTTCATCTGGACTGCCCCAGAAAAATAACTTCAAGCAAACATCCTATCAACAACAAGGTTGTTCT
GCATACCAAGCTGAGCACAGAAGATGGGAACACTGGTGGAGGATGGAAAGGCTCGCTCAATCAAGAAAATTCTGA
GACTATTAATAAATAAGACTGTAGTGTAGATACTGAGTAAATCCATGCACCTAAAC**CTTTT**GGAAAATCTGCCGT
GGGCCCTCCAGATAGCTCATTTCATTAAGTTTTTCCCTCCAAGGTAGAATTTGCAAGAGTGACAGTGGATTGCAT
TT**CTTTT**GGGGAAGCTTT**CTTTT**GGTGGTTTTGTTTATTATACCTTCTTAAGTTTTCAACCAAGGTTTG**CTTTT**G
TTTTGAGTTACTGGGGTTATTTTTGTTTTAAATAAAAATAAGTGTACAATAAGTGTTTTTGTATTGAAAG**CTTTT**
GTTATCAAGATTTTCATA**CTTTT**ACCTTCCATGGCT**CTTTT**TAAGATTGATA**CTTTT**AAGAGGTGGCTGATATTC
TGCAACACTGTACACATAAAAAATACGGTAAGGATACTTTACATGGTTAAGGTAAAGTAAGTCTCCAGTTGGCCA
CCATTAGCTATAATGGCACTTTGTTTGTGTTGTTGGAAAAAGTCACATTGCCATTAAACTTTCCTTGTCTGTCTA
GTTAATATTGTGAAGAAAAATAAAGTACAGTGTGAGATACTG

MYC variant 1 (NM_002467.5)

GGAGTTTATTCATAACGCGCTCTCCAAGTATACGTGGCAATGCGTTGCTGGGTTATTTTAATCATTCTAGGCATC
GTTTTCCTCCTTATGCCTCTATCATTCCTCCCTATCTACACTAACATCCCACGCTCTGAACGCGCGCCCATTAAT
ACCCTTCTTTCCTCCACTCTCCCTGGGACTCTTGATCAAAGCGCGGCCCTTTCCCCAGCCTTAGCGAGGCGCCCT
GCAGCCTGGTACGCGCGTGGCGTGGCGGTGGGCGCGCAGTGCGTTCTCGGTGTGGAGGGCAGCTGTTCCGCCTGC
GATGATTTATACTCACAGGACAAGGATGCGGTTTGTCAAACAGTACTGCTACGGAGGAGCAGCAGAGAAAGGGAG
AGGGTTTGAGAGGGAGCAAAAGAAAATGGTAGGCGCGCGTAGTTAATTCATGCGGCTCTCTTACTCTGTTTACAT
CCTAGAGCTAGAGTGCTCGGCTGCCCGGCTGAGTCTCCTCCCCACCTTCCCCACCCTCCCCACCCTCCCCATAAG
CGCCCCTCCCGGGTTCCCAAAGCAGAGGGCGTGGGGGAAAAGAAAAAAGATCCTCTCTCGCTAATCTCCGCCCAC
CGGCCCTTTATAATGCGAGGGTCTGGACGGCTGAGGACCCCCGAGCTGTGCTGCTCGCGGCCGCCACCGCCGGGC
CCCGGCCGTCCCTGGCTCCCCTCCTGCCTCGAGAAGGGCAGGGCTTCTCAGAGGCTTGGCGGGAAAAAGAACGGA
GGGAGGGATCGCGCTGAGTATAAAAGCCGGTTTTCGGGGCTTTATCTAACTCGCTGTAGTAATTCCAGCGAGAGG
CAGAGGGAGCGAGCGGGCGGCCGGCTAGGGTGGAAGAGCCGGGCGAGCAGAGCTGCGCTGCGGGCGTCCTGGGAA
GGGAGATCCGGAGCGAATAGGGGGCTTCGCCTCTGGCCCAGCCCTCCCGCTGATCCCCCAGCCAGCGGTCCGCAA
CCCTTGCCGCATCCACGAAACTTTGCCCATAGCAGCGGGCGGGCACTTTGCACTGGAACTTACAACACCCGAGCA
AGGACGCGACTCTCCCGACGCGGGGAGGCTATTCTGCCCATTTGGGGACACTTCCCCGCCGCTGCCAGGACCCGC
TTCTCTGAAAGGCTCTCCTTGCAGCTGCTTAGACGCTGGATTTTTTTCGGGTAGTGGAAAACCAGCAGCCTCCCG
CGACGATGCCCCTCAACGTTAGCTTCACCAACAGGAACTATGACCTCGACTACGACTCGGTGCAGCCGTATTTCT
ACTGCGACGAGGAGGAGAACTTCTACCAGCAGCAGCAGCAGAGCGAGCTGCAGCCCCCGGCGCCCAGCGAGGATA
TCTGGAAGAAATTCGAGCTGCTGCCCACCCCGCCCCTGTCCCCTAGCCGCCGCTCCGGGCTCTGCTCGCCCTCCT

ACGTTGCGGTCACACCCTTCTCCCTTCGGGGAGACAACGACGGCGGTGGCGGGAGCTTCTCCACGGCCGACCAGC
TGGAGATGGTGACCGAGCTGCTGGGAGGAGACATGGTGAACCAGAGTTTCATCTGCGACCCGGACGACGAGACCT
TCATCAAAAACATCATCATCCAGGACTGTATGTGGAGCGGCTTCTCGGCCGCCGCCAAGCTCGTCTCAGAGAAGC
TGGCCTCCTACCAGGCTGCGCGCAAAGACAGCGGCAGCCCGAACCCCGCCCGCGGCCACAGCGTCTGCTCCACCT
CCAGCTTGTACCTGCAGGATCTGAGCGCCGCCGCCTCAGAGTGCATCGACCCCTCGGTGGTCTTCCCCTACCCTC
TCAACGACAGCAGCTCGCCCAAGTCCTGCGCCTCGCAAGACTCCAGCGCCTTCTCTCCGTCCTCGGATTCTCTGC
TCTCCTCGACGGAGTCCTCCCCGCAGGGCAGCCCCGAGCCCCTGGTGCTCCATGAGGAGACACCGCCCACCACCA
GCAGCGACTCTGAGGAGGAACAAGAAGATGAGGAAGAAATCGATGTTGTTTCTGTGGAAAAGAGGCAGGCTCCTG
GCAAAAGGTCAGAGTCTGGATCACCTTCTGCTGGAGGCCACAGCAAACCTCCTCACAGCCCACTGGTCCTCAAGA
GGTGCCACGTCTCCACACATCAGCACAACTACGCAGCGCCTCCCTCCACTCGGAAGGACTATCCTGCTGCCAAGA
GGGTCAAGTTGGACAGTGTCAGAGTCCTGAGACAGATCAGCAACAACCGAAAATGCACCAGCCCCAGGTCCTCGG
ACACCGAGGAGAATGTCAAGAGGCGAACACACAACGTCTTGGAGCGCCAGAGGAGGAACGAGCTAAAACGGAG**CT**
TTTTTGCCCTGCGTGACCAGATCCCGGAGTTGGAAAACAATGAAAAGGCCCCCAAGGTAGTTATCCTTAAAAAAG
CCACAGCATACATCCTGTCCGTCCAAGCAGAGGAGCAAAAGCTCATTTCTGAAGAGGACTTGTTGCGGAAACGAC
GAGAACAGTTGAAACACAAACTTGAACAGCTACGGAACTCTTGTGCGTAAGGAAAAGTAAGGAAAACGATTCCTT
CTAACAGAAATGTCCTGAGCAATCACCTATGAACTTGTTTCAAATGCATGATCAAATGCAACCTCACAACCTTGG
CTGAGTCTTGAGACTGAAAGATTTAGCCATAATGTAAACTGCCTCAAATTGGACTTTGGGCATAAAAGAA**CTTTT**
TTATGCTTACCAT**CTTTT**TTTTTTCTTTAACAGATTTGTATTTAAGAATTGTTTTTAAAAAATTTTAAGATTTAC
ACAATGTTTCTCTGTAAATATTGCCATTAAATGTAAATAACTTTAATAAAACGTTTATAGCAGTTACACAGAATT
TCAATCCTAGTATATAGTACCTAGTATTATAGGTACTATAAACCCTAATTTTTTTTATTTAAGTACATTTTG**CTT**
TTTAAAGTTGATTTTTTTCTATTGTTTTTAGAAAAAATAAAATAACTGGCAAATATATCATTGAGCCAAATCTTA
AGTTGTGAATGTTTTGTTTCGTTTCTTCCCCCTCCCAACCACCACCATCCCTGTTTGTTTTCATCAATTGCCCCT
TCAGAGGGTGGTCTTAAGAAAGGCAAGAGTTTTCCTCTGTTGAAATGGGTCTGGGGGCCTTAAGGTCTTTAAGTT
CTTGGAGGTTCTAAGATGCTTCCTGGAGACTATGATAACAGCCAGAGTTGACAGTTAGAAGGAATGGCAGAAGGC
AGGTGAGAAGGTGAGAGGTAGGCAAAGGAGATACAAGAGGTCAAAGGTAGCAGTTAAGTACACAAAGAGGCATAA
GGACTGGGGAGTTGGGAGGAAGGTGAGGAAGAAACTCCTGTTACTTTAGTTAACCAGTGCCAGTCCCCTGCTCAC
TCCAAACCCAGGAATTCTGCCCAGTTGATGGGGACACGGTGGGAACCAGCTTCTGCTGCCTTCACAACCAGGCGC
CAGTCCTGTCCATGGGTTATCTCGCAAACCCCAGAGGATCTCTGGGAGGAATGCTACTATTAACCCTATTTCACA
AACAAGGAAATAGAAGAGCTCAAAGAGGTTATGTAACTTATCTGTAGCCACGCAGATAATACAAAGCAGCAATCT
GGACCCATTCTGTTCAAAACACTTAACCCTTCGCTATCATGCCTTGGTTCATCTGGGTCTAATGTGCTGAGATCA
AGAAGGTTTAGGACCTAATGGACAGACTCAAGTCATAACAATGCTAAGCTCTATTTGTGTCCCAAGCACTCCTAA
GCATTTTATCCCTAACTCTACATCAACCCCATGAAGGAGATACTGTTGATTTCCCCATATTAGAAGTAGAGAGGG
AAGCTGAGGCACACAAAGACTCATCCACATGCCCAAGATTCACTGATAGGGAAAAGTGGAAGCGAGATTTGAACC
CAGGCTGTTTACTCCTAACCTGTCCAAGCCACCTCTCAGACGACGGTAGGAATCAGCTGGCTGCTTGTGAGTACA
GGAGTTACAGTCCAGTGGGTTATGTTTTTTAAGTCTCAACATCTAAGCCTGGTCAGGCATCAGTTCCC**CTTTT**TT
TGTGATTTATTTTGTTTTTATTTTGTTGTTCATTGTTTAATTTTTC**CTTTT**ACAATGAGAAGGTCACCATCTTGA
CTCCTACCTTAGCCATTTGTTGAATCAGACTCATGACGGCTCCTGGGAAGAAGCCAGTTCAGATCATAAAATAAA
ACATATTTATTCTTTGTCATGGGAGTCATTATTTTAGAAACTACAAACTCTCCTTGCTTCCATC**CTTTT**TTACAT
ACTCATGACACATGCTCATCCTGAGTCCTTGAAAAGGTATTTTTGAACATGTGTATTAATTATAAGCCTCTGAAA
ACCTATGGCCCAAACCAGAAATGATGTTGATTATATAGGTAAATGAAGGATGCTATTGCTGTTCTAATTACCTCA
TTGTCTCAGTCTCAAAGTAGGTCTTCAGCTCCCTGTACTTTGGGATTTTAATCTACCACCACCCATAAATCAATA
AATAATTACTTTCTTTGA

VEGFA variant 1 (NM_001171623.1)

TCGCGGAGGCTTGGGGCAGCCGGGTAGCTCGGAGGTCGTGGCGCTGGGGGCTAGCACCAGCGCTCTGTCGGGAGG
CGCAGCGGTTAGGTGGACCGGTCAGCGGACTCACCGGCCAGGGCGCTCGGTGCTGGAATTTGATATTCATTGATC
CGGGTTTTATCCCTCTT**CTTTT**TTCTTAAACATTTTTTTTTAAAACTGTATTGTTTCTCGTTTTAATTTATTTTT
GCTTGCCATTCCCCACTTGAATCGGGCCGACGGCTTGGGGAGATTGCTCTACTTCCCCAAATCACTGTGGATTTT
GGAAACCAGCAGAAAGAGGAAAGAGGTAGCAAGAGCTCCAGAGAGAAGTCGAGGAAGAGAGAGACGGGGTCAGAG
AGAGCGCGCGGGCGTGCGAGCAGCGAAAGCGACAGGGGCAAAGTGAGTGACCTG**CTTTT**GGGGGTGACCGCCGGA
GCGCGGCGTGAGCCCTCCCCCTTGGGATCCCGCAGCTGACCAGTCGCGCTGACGGACAGACAGACAGACACCGCC
CCCAGCCCCAGCTACCACCTCCTCCCCGGCCGGCGGCGGACAGTGGACGCGGCGGCGAGCCGCGGGCAGGGGCCG
GAGCCCGCGCCCGGAGGCGGGGTGGAGGGGGTCGGGGCTCGCGGCGTCGCACTGAAA**CTTTT**CGTCCAACTTCTG
GGCTGTTCTCGCTTCGGAGGAGCCGTGGTCCGCGCGGGGGAAGCCGAGCCGAGCGGAGCCGCGAGAAGTGCTAGC
TCGGGCCGGGAGGAGCCGCAGCCGGAGGAGGGGGAGGAGGAAGAAGAGAAGGAAGAGGAGAGGGGGCCGCAGTGG
CGACTCGGCGCTCGGAAGCCGGGCTCATGGACGGGTGAGGCGGCGGTGTGCGCAGACAGTGCTCCAGCCGCGCGC
GCTCCCCAGGCCCTGGCCCGGGCCTCGGGCCGGGGAGGAAGAGTAGCTCGCCGAGGCGCCGAGGAGAGCGGGCCG
CCCCACAGCCCGAGCCGGAGAGGGAGCGCGAGCCGCGCCGGCCCCGGTCGGGCCTCCGAAACCATGAACTTTCTG
CTGTCTTGGGTGCATTGGAGCCTTGCCTTGCTGCTCTACCTCCACCATGCCAAGTGGTCCCAGGCTGCACCCATG
GCAGAAGGAGGAGGGCAGAATCATCACGAAGTGGTGAAGTTCATGGATGTCTATCAGCGCAGCTACTGCCATCCA

ATCGAGACCCTGGTGGACATCTTCCAGGAGTACCCTGATGAGATCGAGTACATCTTCAAGCCATCCTGTGTGCCC
CTGATGCGATGCGGGGGCTGCTGCAATGACGAGGGCCTGGAGTGTGTGCCCACTGAGGAGTCCAACATCACCATG
CAGATTATGCGGATCAAACCTCACCAAGGCCAGCACATAGGAGAGATGAGCTTCCTACAGCACAACAAATGTGAA
TGCAGACCAAAGAAAGATAGAGCAAGACAAGAAAAAAAATCAGTTCGAGGAAAGGGAAAGGGGCAAAAACGAAAG
CGCAAGAAATCCCGGTATAAGTCCTGGAGCGTGTACGTTGGTGCCCGCTGCTGTCTAATGCCCTGGAGCCTCCCT
GGCCCCCATCCCTGTGGGCCTTGCTCAGAGCGGAGAAAGCATTTGTTTGTACAAGATCCGCAGACGTGTAAATGT
TCCTGCAAAAACACAGACTCGCGTTGCAAGGCGAGGCAGCTTGAGTTAAACGAACGTACTTGCAGATGTGACAAG
CCGAGGCGGTGAGCCGGGCAGGAGGAAGGAGCCTCCCTCAGGGTTTCGGGAACCAGATCTCTCACCAGGAAAGAC
TGATACAGAACGATCGATACAGAAACCACGCTGCCGCCACCACACCATCACCATCGACAGAACAGTCCTTAATCC
AGAAACCTGAAATGAAGGAAGAGGAGACTCTGCGCAGAGCACTTTGGGTCCGGAGGGCGAGACTCCGGCGGAAGC
ATTCCCGGGCGGGTGACCCAGCACGGTCCCTCTTGGAATTGGATTCGCCATTTTATTTTTCTTGCTGCTAAATCA
CCGAGCCCGGAAGATTAGAGAGTTTTATTTCTGGGATTCCTGTAGACACACCCACCCACATACATACATTTATAT
ATATATATATTATATATATATATAAAAATAAATATCTCTATTTTATATATATAAAATATATATATT**CTTTT**TTTAAA
TTAACAGTGCTAATGTTATTGGTGTCTTCACTGGATGTATTTGACTGCTGTGGACTTGAGTTGGGAGGGGAATGT
TCCCACTCAGATCCTGACAGGGAAGAGGAGGAGATGAGAGACTCTGGCATGAT**CTTTT**TTTTGTCCCACTTGGTG
GGGCCAGGGTCCTCTCCCCTGCCCAGGAATGTGCAAGGCCAGGGCATGGGGGCAAATATGACCCAGTTTTGGGAA
CACCGACAAACCCAGCCCTGGCGCTGAGCCTCTCTACCCCAGGTCAGACGGACAGAAAGACAGATCACAGGTACA
GGGATGAGGACACCGGCTCTGACCAGGAGTTTGGGGAGCTTCAGGACATTGCTGTGCTTTGGGGATTCCCTCCAC
ATGCTGCACGCGCATCTCGCCCCCAGGGGCACTGCCTGGAAGATTCAGGAGCCTGGGCGGCCTTCGCTTACTCTC
ACCTGCTTCTGAGTTGCCCAGGAGACCACTGGCAGATGTCCCGGCGAAGAGAAGAGACACATTGTTGGAAGAAGC
AGCCCATGACAGCTCCCCTTCCTGGGACTCGCCCTCATCCTCTTCCTGCTCCCCTTCCTGGGGTGCAGCCTAAAA
GGACCTATGTCCTCACACCATTGAAACCACTAGTTCTGTCCCCCCAGGAGACCTGGTTGTGTGTGTGTGAGTGGT
TGACCTTCCTCCATCCCCTGGTCCTTCCCTTCCCTTCCCGAGGCACAGAGAGACAGGGCAGGATCCACGTGCCCA
TTGTGGAGGCAGAGAAAAGAGAAAGTGTTTATATACGGTACTTATTTAATATCC**CTTTT**TAATTAGAAATTAAA
ACAGTTAATTTAATTAAAGAGTAGGGTTTTTTTTCAGTATTCTTGGTTAATATTTAATTTCAACTATTTATGAGA
TGTAT**CTTTT**GCTCTCTCTTGCTCTCTTATTTGTACCGGTTTTTGTATATAAAATTCATGTTTCCAATCTCTCTC
TCCCTGATCGGTGACAGTCACTAGCTTATCTTGAACAGATATTTAATTTTGCTAACACTCAGCTCTGCCCTCCCC
GATCCCCTGGCTCCCCAGCACACATTCCTTTGAAATAAGGTTTCAATATACATCTACATACTATATATATATTTG
GCAACTTGTATTTGTGTGTATATATATATATATATGTTTATGTATATATGTGATTCTGATAAAATAGACATTGCT
ATTCTGTTTTTTATATGTAAAACAAAACAAGAAAAAATAGAGAATTCTACATACTAAATCTCTCTC**CTTTT**TTA
ATTTTAATATTTGTTATCATTTATTTATTGGTGCTACTGTTTATCCGTAATAATTGTGGGAAAAGATATTAACA
TCACGTCTTTGTCTCTAGTGCAGTTTTTCGAGATATTCCGTAGTACATATTTATTTTTAAACAACGACAAAGAAA
TACAGATATATCTTAAAAAAAAAAAAAGCATTTTGTATTAAAGAATTTAATTCTGATCTCAAAAAAAAAAAAAAAA
AA

IGF2BP1 variant 1 (NM_006546.3)

ATTTAGAGGCGGCGCCAGGGCGGCCGCGGAGAAACGTGACACACCAGCCCTCTCGGAGGGGTTTCGGACCGAAGG
GAAGAAGCTGCGCCGTGTCGTCCGTCTCCCTGCGCGCCGCGGGCACTTCTCCTGGGCTCTCCCCGAACTCTCCCG
CGACCTCTGCGCGCCCTCAGGCCGCCTTCCCCGCCCTGGGCTCGGGACAACTTCTGGGGTGGGGTGCAAAGAAAG
TTTGCGGCTCCTGCCGCCGGCCTCTCCGCCTCTTGGCCTAGGAGGCTCGCCGCCCGCGCCCGCTCGTTCGGCCTT
GCCCGGGACCGCGTCCTGCCCCGAGACCGCCACCATGAACAAGCTTTACATCGGCAACCTCAACGAGAGCGTGAC
CCCCGCGGACTTGGAGAAAGTGTTTGCGGAGCACAAGATCTCCTACAGCGGCCAGTTCTTGGTCAAATCCGGCTA
CGCCTTCGTGGACTGCCCGGACGAGCACTGGGCGATGAAGGCCATCGAAACTTTCTCCGGGAAAGTAGAATTACA
AGGAAAACGCTTAGAGATTGAACATTCGGTGCCCAAAAAACAAAGGAGCCGGAAAATTCAAATCCGAAATATTCC
ACCCCAGCTCCGATGGGAAGTACTGGACAGCCTGCTGGCTCAGTATGGTACAGTAGAGAACTGTGAGCAAGTGAA
CACCGAGAGTGAGACGGCAGTGGTGAATGTCACCTATTCCAACCGGGAGCAGACCAGGCAAGCCATCATGAAGCT
GAATGGCCACCAGTTGGAGAACCATGCCCTGAAGGTCTCCTACATCCCCGATGAGCAGATAGCACAGGGACCTGA
GAATGGGCGCCGAGGGGGCTTTGGCTCTCGGGGTCAGCCCCGCCAGGGCTCACCTGTGGCAGCGGGGGCCCCAGC
CAAGCAGCAGCAAGTGGACATCCCCCTTCGGCTCCTGGTGCCCACCCAGTATGTGGGTGCCATTATTGGCAAGGA
GGGGGCCACCATCCGCAACATCACAAAACAGACCCAGTCCAAGATAGACGTGCATAGGAAGGAGAACGCAGGTGC
AGCTGAAAAAGCCATCAGTGTGCACTCCACCCCTGAGGGCTGCTCCTCCGCTTGTAAGATGATCTTGGAGATTAT
GCATAAAGAGGCTAAGGACACCAAAACGGCTGACGAGGTTCCCCTGAAGATCCTGGCCCATAATAACTTTGTAGG
GCGTCTCATTGGCAAGGAAGGACGGAACCTGAAGAAGGTAGAGCAAGATACCGAGACAAAAATCACCATCTCCTC
GTTGCAAGACCTTACCCTTTACAACCCTGAGAGGACCATCACTGTGAAGGGGGCCATCGAGAATTGTTGCAGGGC
CGAGCAGGAAATAATGAAGAAAGTTCGGGAGGCCTATGAGAATGATGTGGCTGCCATGAGCCTGCAGTCTCACCT
GATCCCTGGCCTGAACCTGGCTGCTGTAGGT**CTTTT**CCCAGCTTCATCCAGCGCAGTCCCGCCGCCTCCCAGCAG
CGTTACTGGGGCTGCTCCCTATAGCTCCTTTATGCAGGCTCCCGAGCAGGAGATGGTGCAGGTGTTTATCCCCGC
CCAGGCAGTGGGCGCCATCATCGGCAAGAAGGGGCAGCACATCAAACAGCTCTCCCGGTTTGCCAGCGCCTCCAT
CAAGATTGCACCACCCGAAACACCTGACTCCAAAGTTCGTATGGTTATCATCACTGGACCGCCAGAGGCCCAATT

CAAGGCTCAGGGAAGAATCTATGGCAAACTCAAGGAGGAGAACTTCTTTGGTCCCAAGGAGGAAGTGAAGCTGGA
GACCCACATACGTGTGCCAGCATCAGCAGCTGGCCGGGTCATTGGCAAAGGTGGAAAAACGGTGAACGAGTTGCA
GAATTTGACGGCAGCTGAGGTGGTAGTACCAAGAGACCAGACCCCTGATGAGAACGACCAGGTCATCGTGAAAAT
CATCGGACATTTCTATGCCAGTCAGATGGCTCAACGGAAGATCCGAGACATCCTGGCCCAGGTTAAGCAGCAGCA
TCAGAAGGGACAGAGTAACCAGGCCCAGGCACGGAGGAAGTGACCAGCCCCTCCCTGTCCCTTCGAGTCCAGGAC
AACAACGGGCAGAAATCGAGAGTGTGCTCTCCCCGGCAGGCCTGAGAATGAGTGGGAATCCGGGACACCTGGGCC
GGGCTGTAGATCAGGTTTGCCCACTTGATTGAGAAAGATGTTCCAGTGAGGAACCCTGATCTCTCAGCCCCAAAC
ACCCACCCAATTGGCCCAACACTGTCTGCCCCTCGGGGTGTCAGAAATTCTAGCGCAAGGCA**CTTTT**AAACGTGG
ATTGTTTAAAGAAGCTCTCCAGGCCCCACCAAGAGGGTGGATCACACCTCAGTGGGAAGAAAAATAAAATTTCCT
TCAGGTTTTAAAAACATGCAGAGAGGTGTTTTAATCAGCCTTAAAGGATGGTTCATTTCTTGACCTTAATGTTTT
TCCAATCTTCTTCCCCCTACTTGGGTAATTGATTAAAATACCTCCATTTACGGCCTCTTTCTATATTTACACTAA
TTTTTTTATCTTTATTGCTACCAGAAAAAAATGCGAACGAATGCATTGCTTTGCTTACAGTATTGACTCAAGGGA
AAAGAACTGTCAGTATCTGTAGATTAATTCCAATCACTCCCTAACCAATAGGTACAATACGGAATGAAGAAGAGG
GGAAAATGGGGAGAAAGATGGTTAAAATACATAATAATCCACGTTTAAAAGGAGCGCACTTGTGGCTGATCTATG
CCAGATCACCATCTTCAAATTGGCACAACTGAAATTTCCCCACTCTGTTGGGGCTTCCCCACCACATTCATGTCC
CTCTCCCGTGTAGGTTTCACATTATGTCCAGGTGCACATAGGTGGTATTGAATGCTCAGCAGGGTAGGGGCTGAC
CACTGTCCCTGATTCCCATCGTTCTCAGGCGGATTTTATATTTTTTTAAAGTCTATTTTAATGATTGGATATGAG
CACTGGGAAGGGGACGCTAACTCCCCTTGATAAAGTCTCGGTTCCATGGAGGACTTGAGTGGCCCCAAAGGCTGC
CACGGTGCCCTCACCCCAGCCCATGTGCTCCCATAAGGGCTGGTTCCTAGAGGCAGGGGTTGTGGGGCACTCCCA
GCCACGGCACTGTTACCTTGGTGGTGGGACTTGGAACCCAACCCTGAGCTCCCGATAAAGCTAAAGTCCATCATC
TGGCAAATTCAGTAAATTGGAGAGTACTTGCTTCTGTTTGTATCTGAGAGGAATTTTTAACTGACGGCTTCTGTC
TCCATGAATCATTATCAGCATGATGAAAGGTGTGTCTAAAAAACAATTCAGAATACCAGCAGCATTGTACAGCAA
GGGGTAAATAAGCTTAATTTATTAATTTACCAGGCTTAATTAAGATCCCATGGAGTGTTTAGCCCTTGTGGGAGA
CAGAAGCCATCAGTTAAATGAGGTTAGGCCTCTCCTCCTAATATACTGATTGACAATGCATATTAGCCAGGTAAT
GCACTTTAGCTACCCTGGACAATGCTATCAAGTGTGCTGGGAAGGGAGGAAGGCCTCTCTACATATGGAAAAGCC
CATGCGTGGAGTTCCCCTCCTTTCAACATTGCAACAACAGTAACAACAAGACAACCGCAACATGTGGGCGTAGTC
AGGCAATGCTGTGTGCGAAGTAAACTACCTCAAGGTATGAAGTTACCTCAGCAATTATTTTC**CTTTT**TGTTCCCC
CCAACCCCATTAAAAAAATTTTTTTTTGATTTTTGTTTTTTTGCAGCTTGCTGATATTTTATATAAAAAAGAAAA
GCAAAGCAAAAGAGAAGCTGATAGTCTTGAATATTTTATTTTTTTAATGAAAAGAAAAAACAAGAAAGTTATGTT
TCATAATTTCTTACAACATGAGCCAGTAACCCTTTAGGAACTCTCTATGGAGAACAGGCCTGGTGGGAAAGGCTT
TGGGGGCTGCCCCCTTAGGAGGAGGCTAGTGCTAAGAGGGAAGGCCCAGGTTTGAGAGAGCCCAGAGGGGCAGAG
CCCAGAGCCTTGTTTGGCCCTGATCTCTGACTTCTAGAGCCCCAGCTGCTGGCGGCTGCTGGAATATCCTACCTG
ATAGGATTAAAAGGCCTAGTGGAGCTGGGGGCTCTCAGTGGTTAAACAATGCCCAACAACCAACCAGCTGGCCCT
TGGTCTCCTCTCTTTCCTCCTTTGGTTAAAGAGCATCTCAGCCAG**CTTTT**CCCACCAGTGGTGCTGTTGAGATAT
TTTAAAATATTGCCTCCGTTTTATCGAGGAGAGAAATAATAACTAAAAAATATACCCTTTAAAAAAACCTATATT
TCTCTGTCTAAAAATATGGGAGCTGAGATTCCGTTCGTGGAAAAAAGACAAGGCCACCCTCTCGCCCTCAGAGAG
GTCCACCTGGTTTGTCATTGCAATG**CTTTT**CATTTTTTTTTTTTGTTATTGTTTCATTTCAGTTCCGTCTTGCTA
TTCTTCCTAATCTATATCCATAGATCTAAGGGGCAAACAGATACTAGTTAACTGCCCCCACCTCTGTCTCCCTGT
CTTCTTTAGATCGGTCTGATTGATTTTAAAAGTGGACCCAAACTTAGGGAATTCTTGATTTAGGGTGGCTGGTGG
CAAGGAGGGGCAGGGGATATGGGGACGTGACTGGGACAGGTTCCTGCCTTATCATTTTCTCCCTAGGACATTCCC
TTGTAGCCCCCAGAATTGTCTGGCCCAAATTGAATAGAAGCAGAAAAACATTTAGGGATAACATCAGGCCAGTAG
AATTAAGCCTCTCCACCTGTCCCAACCATAAAAAGGGTCTCCCAGCTTTCCATCTCTGGCTCTATATGCTTTATC
CCAAAACAAAGCAGATAACGTTCAGACGTCGGCCATTTAGTAATTTAAAGCGAATTTCCAGCAGCAAGCATGCTT
TGATATCTGGTTCAGACTATCATCAGGAAGAAAAAAAAATCCCACAGTACCTGAAATGTGATTGTTGCAGTGTTC
AGTTTCCTTGGGGGCCTGCTCCCTTCACACCTTGAGCCCAAGTC**CTTTT**CCGTTGGCTGATTCAGCTCCCAGAAG
AGACGAGGAAGTGTGTGGCAAGGGACTGGAAAACTTCACTTGCTTGGATTAGGCAAGGCTCCACTCATTGTTGAT
ATTTGCCCAGCAGGAAAATCATGTAAGTTATACCACCAGAAAGCAAAAGGAGCATGGTTTGGTGGTTAAGGTTTA
GTGGGATGAAGGACCTGTCTTGGTGGGCCGGGCCCTCTTGTGCCCCGTAGGCTAGGTCTTAGGGCAACTCCTTGC
CCTCCTGCTCAGCACCTCCATTTCCCCATCCTTGGTGAGATAACAAGCTATCGCGAAAAGCACTTGGGAGATTTG
GATGATTTGAGAAGAGTGACTTAAAAAAAATGCTTCTGTGCTCTAAGATATATATGTGTGTGTGTGTGCTACATA
TATATTTTTAAGAAAGGACCATCTCTTTAGGATATATTTTTAAATTCTTTGAAACACATAACCAAAATGGTTTGA
TTCACTGACTGACTTTGAAGCTGCATCTGCCAGTTACACCCCAAATGGCTTTAATCCCCTCTCGGGTCTGGTTGC
CTTTTGCAGTTTGGGTTGTGGACTCAGCTCCTGTGAGGGGTCTGGTTAGGAGAGAGCCATTTTTAAGGACAGGGA
GTTTTATAGCC**CTTTT**CTACTTTCCTCCCCTCCTCCCAGTCCTTATCAAT**CTTTT**TTC**CTTTT**TCCTGACCCCCT
CCTTCTGGAGGCAGTTGGGAGCTATCCTTGTTTATGCCTCACTATTGGCAGAAAAGACCCCATTTAAAACCCAGA
GAACACTGGAGGGGGATGCTCTAGTTGGTTCTGTGTCCATTTTCCTCTGTGCCAAAGACAGACAGACAGAGGCTG
AGAGAGGCTGTTCCTGAATCAAAGCAATAGCCAGCTTTCGACACATACCTGGCTGTCTGAGGAGGAAGGCCTCCT
GGAAACTGGGAGCTAAGGGCGAGGCCCTTCCCTTCAGAGGCTCCTGGGGGATTAGGGTGTGGTGTTTGCCAAGCC
AAGGGGTAGGGAGCCGAGAAATTGGTCTGTCGGCTCCTGGTTGCACTTTGGGGAAGGAGAGGAAGTTTGGGGCTC
CAGGTAGCTCCCTGTTGTGGGACTGCTCTGTCCCCTGCCCCTACTGCAGAGATAGCACTGCCGAGTTCCCTTCAG
GCCTGGCAGACGGGCAGTGAGGAGGGGCCTCAGTTAGCTCTCAAGGGTGCCTTCCCCTCCTCCCAACCCAGACAT
ACCCTCTGCCAAACTGGGAACCAGCAGTGCTAGTAACTACCTCACAGAGCCCCAGAGGGCCTGCTTGAGCCTTCT

TGCTCCACAGGAGAAGCTGGTGCCTCTAGGCAACCCCTTCCTCCCACCTCTCATCAGGGGTGGGGGTTCTCCTTT
CTTTCCCCTGAAGTGTTTATGGGGAGATCCTAGTGGCTTTGCCATTCAAACCACTCGACTGTTTGCCTGTTTCTT
GAAAACCAGTAGAAGGGAAACAGCACAGCCTGTCACAGTAATTGCAGGAAGATTGAAGAAAAATCCTCATCAATG
CCAGGGGACATAAAAGCCATTTCCCTTCCAAATACTCGACAATTTAGATGCAGAACATTTCTCTGTATTCAGACT
TAGAGTAACACCAGCTGAAAACTGCAGTTTCTTTCCTTTGGATACATAAGGCTTCTCTATCGGGGTACGGACAG
GGAGGAGGCCTCATGTCTGAAGGGGGATTTAGGGGCGAGAGCCCCAGCCCTGACCCTCGGTCCTGTGCACCGCTT
TGGGGCACAGTCTGATGGCGCCTTTGCTGGCGCCTTAGTATGGTTGACTCCGGATGGACAAAAGAAAAAAAATTT
TTTTTCTTGAATGAAATAGCAGGAAGCTCCTCGGGAGCATGTGTTTTGATTAACCGCAGGTGATGGATGCTACGA
GTATAAATGGATTAACTACCTCAATCCTTACAGTAAGATTGGAACTAAGGGCAGGGACTCATGCATAAGGGTATG
AATCCCAGCCAGGACAAGTGAGTTGAGGCTTGTGCCACAAAAGGTTTGTCCTTGGGGAACAGGCAGGCCTGCCAG
GATCCCCCCCATATCGATTGGGCTGGGAGGGCTGGCCATGAGGTCCCCACTTTCTGCTTTCCTTGCCCATGTGTC
ACCCCTTTGGCCTCCAGCTTGTCCCTCTCTCACTTTCTATAGCTTTGTTGGACCAGATGGTGAGGAAAGGAATGG
CCTCTTCCCTTCTAGAGGGGGCTGGCTGGAGTGAGACCTGGGGCTTGGCCTGGAACCCACCACACAGCCCCAAAG
TCAGGAAGCCTGGGGAAACCAGAGCTGAGACCTCTTCAACAGGGTTTCTTTGAGATCCTACACCTCCATTGGGCC
CTTTTTCAGTCTTCAATGGGGGCCCAGTTGGCTCTAGAAGGAGAAGAGGTGAAGCAGGATCCTTTGCCCTGGGGG
AGTCTGAGGGCGCGGTCCTTGGACTCATTCAGGCCGTCTTTGTAGTTGGGGGAGTTCCACTGGGCGATCCCAGCC
CCTCCCCACCCACCCTCTAATGGACCTCCTCATAGAAGCCCCATTTCA**CTTTT**GTTTTATCTACCTCTTAGCAAA
ACAATAGATAAATTAGGTAGTGGCAGCTCCACTTGCTTAGGTTAGGGGGGGAAAAAGATTT**CTTTT**TCCAAAGGA
AAAAAATATTACCTTGAGAATACTTTCCAAAAAATAAAATTAAAAAAAAAAAAACCAAAAAAAAAAATTTTTTTT
TAAAAGGGAGACATTTTCCAGTGACCACTGGATTGTTTTAATTTCCCAAG**CTTTT**TTTTCCCCCATAAATAAGTT
TCACTCTTTGGCGATTTTCTTCACTTGTTTAAGATAACGTGCTAGCTATTCCAACAGGTAACAGCTTTCACAGTC
TGCCCCTGGCCTGTCTCACCCCATCCCCCACCCTATTCCTGCCAGTGAGTCCTTCCTGTGCTTCTCTCCCTTCTC
CCCTCCCAGCCAGCTGACTTCAGTCACCCCTGTCCCCCCTCCCCTGCCAATAAGCTCCCCCAGGAATAAAGGCTT
TGTTTTGGGGATGCTTAAATCTTGACTGGCACTTCCCGGCTGTGGGGGCTGGGGAGCCACTTGTAACATTTCTGT
GCAGATTTTATGTTAGCCACTGCTATGTAAAAGCACGTTCAAAATGAATTTCAGCAGATTATGTGTTACCATAAT
GAATAAACGTCCTCTATCACCATTTGGAGTCTCC**CTTTT**CTCCAGGATCTTGATCCTGGTCCCCAAAACCAGAGT
GAATCAAAAGAGCTTCCTCCCCTGAGGCAAAGTGGATTTGTAAGCAGTTCTGAAACATCACTTACTCAGAAGAGG
GAACGATGTATTTTGATGAGTGCAAATTGGGAAGAGCTGGAGGCCTACTGCTTGGGACAGTTTTTTTTTTTTTTT
TTTTTTTAAATATGAGTGCTAGCTTATTCTGTAATTGCGGCAACTTTGAAAATTGTATTTTACTGGAAATCTGCC
AGCCATCACCACCCGATTTTGATTGTATCCTTCCTCCCATCCTTTAATCTGTTCATTGCTTTGGGGGAGGTGGGG
CAGCTGGCTCACACGTTGGAGTTTGTTCTTTGATGGATGAACGAACACTCCAGTTTTCTTTCCCGTGAAGGTTGT
TTCAGCCACAAACCACTTCATTTTGCTGTTTCAATTTCAAAATAAAAGGAAACTTATATTGAAAGACAA

IGF2BP3 (NM_006547.2)

AAGACTTAGGAAGACTGGTGGATGCGTTTGGGTTGTAGCTAGG**CTTTT**T**CTTTT**CTTTCT**CTTTT**AAAACACATC
TAGACAAGGAAAAAACAAGCCTCGGATCTGATTTTTCACTCCTCGTTCTTGTGCTTGGTTCTTACTGTGTTTGTG
TATTTTAAAGGCGAGAAGACGAGGGGAACAAAACCAGCTGGATCCATCCATCACCGTGGGTGGTTTTAATTTTTC
GTTTTTTCTCGTTATTTTTTTTTAAACAACCACTCTTCACAATGAACAAACTGTATATCGGAAACCTCAGCGAGA
ACGCCGCCCCCTCGGACCTAGAAAGTATCTTCAAGGACGCCAAGATCCCGGTGTCGGGACCCTTCCTGGTGAAGA
CTGGCTACGCGTTCGTGGACTGCCCGGACGAGAGCTGGGCCCTCAAGGCCATCGAGGCGCTTTCAGGTAAAATAG
AACTGCACGGGAAACCCATAGAAGTTGAGCACTCGGTCCCAAAAAGGCAAAGGATTCGGAAACTTCAGATACGAA
ATATCCCGCCTCATTTACAGTGGGAGGTGCTGGATAGTTTACTAGTCCAGTATGGAGTGGTGGAGAGCTGTGAGC
AAGTGAACACTGACTCGGAAACTGCAGTTGTAAATGTAACCTATTCCAGTAAGGACCAAGCTAGACAAGCACTAG
ACAAACTGAATGGATTTCAGTTAGAGAATTTCACCTTGAAAGTAGCCTATATCCCTGATGAAATGGCCGCCCAGC
AAAACCCCTTGCAGCAGCCCCGAGGTCGCCGGGGGCTTGGGCAGAGGGGCTCCTCAAGGCAGGGGTCTCCAGGAT
CCGTATCCAAGCAGAAACCATGTGATTTGCCTCTGCGCCTGCTGGTTCCCACCCAATTTGTTGGAGCCATCATAG
GAAAAGAAGGTGCCACCATTCGGAACATCACCAAACAGACCCAGTCTAAAATCGATGTCCACCGTAAAGAAAATG
CGGGGGCTGCTGAGAAGTCGATTACTATCCTCTCTACTCCTGAAGGCACCTCTGCGGCTTGTAAGTCTATTCTGG
AGATTATGCATAAGGAAGCTCAAGATATAAAATTCACAGAAGAGATCCCCTTGAAGATTTTAGCTCATAATAACT
TTGTTGGACGTCTTATTGGTAAAGAAGGAAGAAATCTTAAAAAAATTGAGCAAGACACAGACACTAAAATCACGA
TATCTCCATTGCAGGAATTGACGCTGTATAATCCAGAACGCACTATTACAGTTAAAGGCAATGTTGAGACATGTG
CCAAAGCTGAGGAGGAGATCATGAAGAAAATCAGGGAGTCTTATGAAAATGATATTGCTTCTATGAATCTTCAAG
CACATTTAATTCCTGGATTAAATCTGAACGCCTTGGGTCTGTTCCCACCCACTTCAGGGATGCCACCTCCCACCT
CAGGGCCCCCTTCAGCCATGACTCCTCCCTACCCGCAGTTTGAGCAATCAGAAACGGAGACTGTTCATCTGTTTA
TCCCAGCTCTATCAGTCGGTGCCATCATCGGCAAGCAGGGCCAGCACATCAAGCAGCTTTCTCGCTTTGCTGGAG
CTTCAATTAAGATTGCTCCAGCGGAAGCACCAGATGCTAAAGTGAGGATGGTGATTATCACTGGACCACCAGAGG
CTCAGTTCAAGGCTCAGGGAAGAATTTATGGAAAAATTAAAGAAGAAAACTTTGTTAGTCCTAAAGAAGAGGTGA
AACTTGAAGCTCATATCAGAGTGCCATCCTTTGCTGCTGGCAGAGTTATTGGAAAAGGAGGCAAAACGGTGAATG
AACTTCAGAATTTGTCAAGTGCAGAAGTTGTTGTCCCTCGTGACCAGACACCTGATGAGAATGACCAAGTGGTTG
TCAAAATAACTGGTCACTTCTATGCTTGCCAGGTTGCCCAGAGAAAAATTCAGGAAATTCTGACTCAGGTAAAGC

AGCACCAACAACAGAAGGCTCTGCAAAGTGGACCACCTCAGTCAAGACGGAAGTAAAGGCTCAGGAAACAGCCCA
CCACAGAGGCAGATGCCAAACCAAAGACAGATTGCTTAACCAACAGATGGGCGCTGACCCCCTATCCAGAATCAC
ATGCACAAGTTTTTACCTAGCCAGTTGTTTCTGAGGACCAGGCAA**CTTTT**GAACTCCTGTCTCTGTGAGAATGTA
TACTTTATGCTCTCTGAAATGTATGACACCCAGCTTTAAAACAAACAAACAAACAAACAAAAAAAGGGTGGGGGA
GGGAGGGAAAGAGAAGAGCTCTGCACTTCCCTTTGTTGTAGTCTCACAGTATAACAGATATTCTAATTCTTCTTA
ATATTCCCCCATAATGCCAGAAATTGGCTTAATGATGCTTTCACTAAATTCATCAAATAGATTGCTCCTAAATCC
AATTGTTAAAATTGGATCAGAATAATTATCACAGGAACTTAAATGTTAAGCCATTAGCATAGAAAAACTGTTCTC
AGTTTTATTTTTACCTAACACTAACATGAGTAACCTAAGGGAAGTGCTGAATGGTGTTGGCAGGGGTATTAAACG
TGCATTTTTACTCAACTACCTCAGGTATTCAGTAATACAATGAAAAGCAAAATTGTTC**CTTTT**TTTTGAAAATTT
TATATACTTTATAATGATAGAAGTCCAACCGTTTTTTAAAAAATAAATTTAAAATTTAACAGCAATCAGCTAACA
GGCAAATTAAGATTTTTACTTCTGGCTGGTGACAGTAAAGCTGGAAAATTAATTTCAGGGTTTTTTGAGG**CTTTT**
GACACAGTTATTAGTTAAATCAAATGTTCAAAAATACGGAGCAGTGCCTAGTATCTGGAGAGCAGCACTACCATT
TATTCTTTCATTTATAGTTGGGAAAGTTTTTGACGGTACTAACAAAGTGGTCGCAGGAGATTTTGGAACGGCTGG
TTTAAATGGCTTCAGGAGACTTCAGTTTTTTGTTTAGCTACATGATTGAATGCATAATAAATGCTTTGTGCTTCT
GACTATCAATACCTAAAGAAAGTGCATCAGTGAAGAGATGCAAGACTTTCAACTGACTGGCAAAAAGCAAGCTTT
AGCTTGTCTTATAGGATGCTTAGTTTGCCACTACACTTCAGACCAATGGGACAGTCATAGATGGTGTGACAGTGT
TTAAACGCAACAAAAGGCTACATTTCCATGGGGCCAGCACTGTCATGAGCCTCACTAAGCTATTTTGAAGATTTT
TAAGCACTGATAAATTAAAAAAAAAAAAATTAGACTCCACCTTAAGTAGTAAAGTATAACAGGATTTCTGTATACT
GTGCAATCAGTTCTTTGAAAAAAAAGTCAAAAGATAGAGAATACAAGAAAAGTTTTTGGGATATAATTTGAATGA
CTGTGAAAACATATGACCTTTGATAACGAACTCATTTGCTCACTCCTTGACAGCAAAGCCCAGTACGTACAATTG
TGTTGGGTGTGGGTGGTCTCCAAGGCCACGCTGCTCTCTGAATTGATTTTTTGAGTTTTGTTTGTAAGATGATCA
CAGTCATGTTACACTGATCTAAAGGACATATATATAACCCTTTAAAAAAAAAATCACTGCCTCATTCTTATTTCA
AGATGAATTTCTATACAGACTAGATGTTTTTCTGAAGATCAATTAGACATTTTGAAAATGATTTAAAGTGTTTTC
CTTAATGTTCTCTGAAAACAAGTTT**CTTTT**GTAGTTTTAACCAAAAAAGTGCC**CTTTT**TGTCACTGGATTCTCCT
AGCATTCATGATTTTTTTTTCATACAATGAATTAAAATTGCTAAAATCATGGACTGGCTTTCTGGTTGGATTTCA
GGTAAGATGTGTTTAAGGCCAGAG**CTTTT**CTCAGTATTTGATTTTTTTCCCCAATATTTGATTTTTTAAAAATAT
ACACATAGGTGCTGCATTTATATCTGCTGGTTTAAATTCTGTCATATTTCACTTCTAGC**CTTTT**AGTATGGCAAA
TCATATTTTA**CTTTT**ACTTAAGCATTTGTAATTTGGAGTATCTGGTACTAGCTAAGAAATAATTCTATAATTGAG
TTTTGTACTCACCATATATGGATCATTCCTCATGTATAATGTGCCCCAAATGCAGCTTCATTTTCCAGATACCTT
GACGCAGAATAAATTTTTTCATCATTTAGGTGCAAAAAAAAAA

NEAT1 variant 2 (NR_131012.1)

GGAGTTAGCGACAGGGAGGGATGCGCGCCTGGGTGTAGTTGTGGGGGAGGAAGTGGCTAGCTCAGGGCTTCAGGG
GACAGACAGGGAGAGATGACTGAGTTAGATGAGACGAGGGGGCGGGCTGGGGGTGCGAGAAGGAAGCTTGGCAAG
GAGACTAGGTCTAGGGGGACCACAGTGGGGCAGGCTGCATGGAAAATATCCGCAGGGTCCCCCAGGCAGAACAGC
CACGCTCCAGGCCAGGCTGTCCCTACTGCCTGGTGGAGGGGGAACTTGACCTCTGGGAGGGCGCCGCTCTTGCAT
AGCTGAGCGAGCCCGGGTGCGCTGGTCTGTGTGGAAGGAGGAAGGCAGGGAGAGGTAGAAGGGGTGGAGGAGTCA
GGAGGAATAGGCCGCAGCAGCCCTGGAAATGATCAGGAAGGCAGGCAGTGGGTGCAGGGCTGCAGGAGGGCCGGG
AGGGCTAATCTTCAACTTGTCCATGCCAGCAGCCC**CTTTTTTT**CCAGACCAAGGGCTGTGAACCCGCCTGGGGAT
GAGGCCTGGTCTTGTGGAACTGAACTTAGCTCGACGGGGCTGACCGCTCTGGCCCAGGGTGGTATGTAATTTTCG
CTCGGCCTGGGACGGGGCCCAGGCCGGGCCCAGCCTGGTGGAGCGTCCAGGTCTGGGTGCGAAGCCAGGCCCCTG
GGCGGAGGTGAGGGGTGGTCTGAGGAGTGATGTGGAGTTAAGGCGCCATCCTCACCGGTGACTGGTGCGGCACCT
AGCATGTTTGACAGGCGGGGACTGCGAGGCACGCTGCTCGGGTGTTGGGGACAACATTGACCAACGCTTTATTTT
CCAGGTGGCAGTGCTC**CTTTT**GGA**C**TTTTCTCTAGGTTTGGCGCTAAACTCTTCTTGTGAGCTCACTCCACCCCT
TCTTCCTCCCTTTAACTTATCCATTCACTTAAAACATTACCTGGTCATCTGGTAAGCCCGGGACAGTAAGCCGAG
TGGCTGTTGGAGTCGGTATTGTTGGTAATGGTGGAGGAAGAGAGGCCTTCCCGCTGAGGCTGGGGTGGGGCGGAT
CGGTGTTGCTTGCCTGCAGAGAGGGTGGGGAGTGAATGTGCACCCTTGGGTGGGCCTGCAGCCATCCAGCTGAAA
GTTACAAAAATGCTTCATGGACCGTGGTTTGTTACTATAGTGTTCCTCATGGCGAGCAGATGGAACCGGGAGACA
TGGAGTCCCTGGCCAGTGTGAGTCCTAGCATTGCAGGAGGGGAGACCCTGGAGGAGAGAGCCCGCCTCAATTGAT
GCCTGCAGATTGAATTTCCAGAGGCTTAGGAGGAGGAAGTTCTCCAATGTTCTGTTTCCAGGCCTTGCTCAGGAA
GCCCTGTATTCAGGAGGCTACCATTTAAAGTTTGCAGATGAGCTTATGGGGGGCAATCTTAAAAAGTCCACAGCA
GATGCATCCGGCTCGAGGGGCCATCAGCTTTGAATAAATGCTTGTTCCAGAGCCCATGAATGCCAGCAGGCACCC
CTCCTTTCCTGGGGTAAAGGTTTTCAGATGCTGCATCTTCTAAATTGAGCCTCCGGTCATACTAGTTTTGTGCTT
GGAACCTTGCTTCAAGAAGATCCCTAAGCTGTAGAACATTTTAACGTTGATGCCACAACGCAGATTGATGCCTTG
TAGATGGAGCTTGCAGATGGAGCCCCGTGACCTCTCACCTACCCACCTGTTTGCCTGCCTTCTTGTGCGTTTCTC
GGAGAAGTTCTTAGCCTGATGAAATAACTTGGGGCGTTGAAGAGCTGTTTAATTTTAAATGCCTTAGACTGGGGA
TATATTAGAGGAAGCAGATTGTCAAATTAAGGGTGTCATTGTGTTGTGCTAAACGCTGGGAGGGTACAAGTTGGT
CATTCCTAAATCTGTGTGTGAGAAATGGCAGGTCTAGTTTGGGCATTGTGATTGCATTGCAGATTACTAGGAGAA

GGGAATGGTGGGTACACCGGTAGTGCT**CTTTT**GTTCTTGCTTCGTTTTTTTAAACTTGAACTTTACTTCGTTAGA
TTTCATAATACTTTCTTGGCATTCTAGTAAGAGGACCCTGAGGTGGGAGTTGTGGGGGACGGGGAGAAGGGGACA
GCTTGGCACCGGTCCCGTGGGCGTTGCAGTGTGGGGGATGGGGGTATGCAGCTTGGCACTGGTACTGGGAGGGAT
GAGGGTGAAGAAGGGGAGAGGGTTGGTTAGAGATACAGTGTGGGTGGTGGGGGTGGTAGGAAATGCAGGTTGAAG
GGAATTCTCTGGGGCTTTGGGGAATTTAGTGCGTGGGTGAGCCAAGAAAATACTAATTAATAATAGTAAGTTGTT
AGTGTTGGTTAAGTTGTTGCTTGGAAGTGAGAAGTTGCTTAGAAACTTTCCAAAGTGCTTAGAACTTTAAGTGCA
AACAGACAAACTAACAAACAAAAATTGTTTTGCTTTGCTACAAGGTGGGGAAGACTGAAGAAGTGTTAACTGAAA
ACAGGTGACACAGAGTCACCAGTTTTCCGAGAACCAAAGGGAGGGGTGTGTGATGCCATCTCACAGGCAGGGGAA
ATGTCTTTACCAGCTTCCTCCTGGTGGCCAAGACAGCCTGTTTCAGAGGGTTGTTTTGTTTGGGGTGTGGGTGTT
ATCAAGTGAATTAGTCACTTGAAAGATGGGCGTCAGACTTGCATACGCAGCAGATCAGCATCCTTCGCTGCCCCT
TAGCAACTTAGGTGGTTGATTTGAAACTGTGAAGGTGTGATTTTTTCAGGAGCTGGAAGTCTTAGAAAAGCCTTG
TAAATGCCTATATTGTGGG**CTTTT**AACGTATTTAAGGGACCACTTAAGACGAGATTAGATGGGCTCTTCTGGATT
TGTTCCTCATTTGTCACAGGTGTCTTGTGATTGAAAATCATGAGCGAAGTGAAATTGCATTGAATTTCAAGGGAA
TTTAGTATGTAAATCGTGCCTTAGAAACACATCTGTTGT**C**TTTTCTGTGTTTGGTCGATATTAATAATGGCAAAA
TTTTTGCCTATCTAGTATCTTCAAATTGTAGTCTTTGTAACAACCAAATAAC**CTTTT**GTGGTCACTGTAAAATTA
ATATTTGGTAGACAGAATCCATGTACCTTTGCTAAGGTTAGAATGAATAATTTATTGTATTTTTAATTTGAATGT
TTGTG**CTTTTT**AAATGAGCCAAGACTAGAGGGGAAACTATCACCTAAAATCAGTTTGGAAAACAAGACCTAAAAA
GGGAAGGGGATGGGGATTGTGGGGAGAGAGTGGGCGAGGTGCCTTTACTACATGTGTGATCTGAAAACCCTGCTT
GGTTCTGAGCTGCGTCTATTGAATTGGTAAAGTAATACCAATGG**CTTTTT**ATCATTTCCTTCTTCCCTTTAAGTT
TCACTTGAAATTTTAAAAATCATGGTTATTTTTATCGTTGGGATCTTTCTGTCTTCTGGGTTCCATTTTTTAAAT
GTTTAAAAATATGTTGACATGGTAGTTCAGTTCTTAACCAATGACTTGGGGATGATGCAAACAATTACTGTCGTT
GGGATTTAGAGTGTATTAGTCACGCATGTATGGGGAAGTAGTCTCGGGTATGCTGTTGTGAAATTGAAACTGTAA
AAGTAGATGGTTGAAAGTACTGGTATGTTGCTCTGTATGGTAAGAACTAATTCTGTTACGTCATGTACATAATTA
CTAATCA**C**TTTTCTTCCCCTTTACAGCACAAATAAAGTTTGAGTTCTAAACTCATTAGAATTGTTGTATTGCTAT
GTTACATTTCTCGACCCCTATCACATTGCCTTCATAACGACTTTGGATGTATCTTCATATTGTAGATTTAGGTCT
AGATTTGCTAGCTCCAAGTAATTAAGGCCATGTAGGAGAGCATGGTAACCACAGATAGAACTGGTATTATCCCAA
GTGGTCTGCAGACTGCTGAGTGGGGATGGGATCTGCTCTCTGTTGAGAGTTGGTAATCATTGGTTTGAAATGTGA
TGAAACCACTCAAGCCAATGAAGGTGGGTGTGTAGGTGGGGAGTACTTTGCCATAATATTTTAAAACATTACCTG
GTTAGAGTTCTAAGTGGTACTTATTTTTGTTTGGTTAGGGGAAAGCCTGAATAAAAACAGAAATGGACACATAAT
ATGCATATTCCATAGTCTTTGGGAGGCTGGAATGTGCCTGGGATTTGGGTCTAAGTGTATGCGTAATTCTTACCT
CACTAAAGAATTTGCCTTGTTTTTTTC**CTTTT**GGTGAGTGACTAAAACGTCTGGGCTTCCCTGTGTGCGTGCTAC
AGTAAGCAAGCAGAGGCTGTGCAAAGGTGTGAGCAGGATCACGTGGAATCTGGAGGATACATCTTGGCTTGCAAA
CTGCCTCTGTCTCCTGGGTGGGACTGTTCTGTCCTTGCACTGCTGTTCTGTGTTACCTCTTGGGGTGTAAGGTTT
TGCTTACAGGAGACAAACTTTGGGCGTAGAATGGAAGCCACTGCCAGCCTCTGTGCTGAGAAGGAAGGTGCTTGT
TTCAAAGGGAGCAGCAAGGGAGGCTTGTTCTACTCACCTGGGCCTGTTTGCCTGAGAAGGGGAGATAAGGGCTGA
ACTGGGACTAGCCAGGGGGACCAACACAAATGGTGGGGGATCATGACCTGAAGGATTCTTTCCTTCCCATGAGCT
GCAGGGCTGGTTGCCGTCCTTGCAACTGTGTCTTATTTGCCTGTGCCGTTATATCTTGGTGACCCCTCCACGTGT
ACACTACTGACAAACGGGTGGAGTGCTGGGGAGAAGTCACTGTGCCGCCCACCTAGTAAACCTTCTGTCTGTGCT
CATGGCATCTCCAAGATGGGGCACTGCTGTGTGCAGAATCCAGGGTCCTCTTTCTGCTTGCAACTCCTTTCCCTG
GATGCCCCAGAAACAATCCAGGCCTCCTTTCCTATCTTACCCCTTTGCTTTG**CTTTTT**ACCCCAGCACCTCTATA
ACCGCCTTCTCTT**C**TTTTCAGAACTCCTTGTTTCTCGTCCTGTTTTTTATGATTACAAAACTCTTGCTTCCACCC
TGGAAGATAACTGCTATAGATGCCTGTATGTAAATGGTGCTGTCTCCAGCAACTGGCATGCTGAAGAAGAATTGA
TTCACGGGGTATAAATGTTGGGGATTGGAAGTGGGGATGAAATGGCACTTGTTGATACAGGAGCAGAGAGGTGAG
GCCGACTGCTGAAGACAGCTCGCCACCCTCCTTGCCTCCACTCCAATCCAGGGGCTGGGGCCACATTCTTTGCCT
TCATTTATCCTCAGATCAGGTGAGATCGACAGGAGGTGTTGATGGCAGTGCCAGCAATTATTGCTAATCCGTTTG
CATCCTTATGCATAGATCTGAATTCAGACTTTGTGAATTTCCAGAGGTGTGGGTAATATAATAGAATTCAGTGAG
TGGGCATGGCTGATCTTGTGCAAATTAAAAGTTATGGGGCATAAGAATAGCAAAAGTTGAACTT**CTTTT**AAAAAG
GAAAGTACCCTGAGAGCCAGTATTGGTTGAGGCTCTTCAGTATGCCCAGGTTGGCAGCACTGAGAACCGCAGGAA
CGGCCTGTTGTTACAAAAAGGAGATTGACTCAGCTGCCCTTGGTGCATCTGACTGACTATGACTGCTGAGAGATT
CCAAGGACCCTTAATGCCAGGGCTAACCTCTCCATGTGCAGTGAGACCTCTGGAGGAAGTGTCATCCTCTGGCTT
TGTGTGGTACTCATTATGGTGCAGTGCGGGCATGAAATGAAGACACCCAAATAGGCTTACAGATACGATATGTTT
TAAATGTTCGTATTTAACAAAAACATACTGACACTGTTTGGAAATGGCAACAGGAAGATAGCAAAATGAATACTA
ACATTACGAAAAGATGAACAGGTACATGTTCCAAGGCAGGTGGCTGTGAACTTCCTCTGAGTGAAGGCATCCCCT
CCAGCACCTTTCAGCCTGCTAGTTAGGACGACCCGCCGCCACCCTCCAGGACCTCCAGCCCTGCACTGCCTTTCC
TCT**CTTTT**AAATAATTCTTCATTGAGTTCTAATATGTAAAAAAAAAAAGTTTACTGTAAAGTTTGCAAATAAGGA
AATTTTTTTTAAAAGTCCTCAGTAATCTTACCAGTAACAATTGTTATGGGCACATTTG**CTTTT**GGAAGATTT**CTT**
TTGTATGCATGGGATAAGTACATTTTTAAACAAAAATGGGATTATGCCATAAATTCTATTTTGTGACTTTAATAT
ATAGTGAACAC**CTTTTT**TAATGATGACAGGATGTTCCCTTGCATGGCTGTATCAATTTAAACAATCTTGTTTCAA
TGGGCATACAGGGTATTTTCTAGTTTTTTTTTCCTCTTAGAAAATAATACTTGCGATGACTTTCCTTGTAGCTCA
GA**C**TTTTTCACGTCTGTTGTTATCTCTTTGGGAATGCTGAATACATACATTTCGAGAAGGAAATGACTGTTAAAC
TCTTAAGACTTCAGGTTCATATTGCTAAACTGCCCAGCAGGGAGGGATTTTTTCAATTAGTGTTCTCACTGGTGA
GGCAAACCTGATGCCTTCCCCTCTTCCTCAGAACCGGCTTTATCACATTGAAAACCTTTGCTCCTCCGACGGATC

GAGTCTGCTTTCCCTCTGGATGTGAGCATTGCTTTGTCTGCTGGTGACTGAACATCTCTACCTTGTGTCAATTGG
CCATTTGTGGTGTGTGTGTGTGTGCGTGTGTGTGTGTGTGTGTGTATGATTTTCTAATTCCTAGTCATTTTTC
TATTGATTGTTTTGCAAAAGCCATTTACATCTTAAGGATATTGATAAT**CTTTT**GTTATATTTGATGCAAATATTT
TTTTCCAGTTTATAGGTTGC**CTTTT**AATTTTGTGTTTCAGGTAGATAAAAGTTAAACGATTTTCTTAGGTTAGTT
TATCACTGTGGTTTCTGAACTTGTTATGTGTAGAT**C**TTTTCCACCCCAAGAGTACATAAATATTAATCCATACTT
TCTTATGGAACTTGTATGGTTTCGTTTTTTACATTTAAACCTTCTTCCCCGTGGTGTGTGTTGTGGAATCTGTGT
TTGTGTGAGGAGGGGCATGGTGCTCTCAGAACCCACCTCCTGTGGCCAGAGAGCCCTGTCCTGTGAGGGTGGTTG
TCACAGTGGCAGGGTTCAATTCAGAAGACCTTGAGGGCAGGCTGATGTTTCCTGAATGGGCCCCTGGTTGTTGCT
TGTCCCTGACTCTCCATTTCCCCATCTGAGTGGATTTGGACCTAATAGGGCACTGGAGCTGGTTCGAATCCTGAC
TGGACTACTTGGCAACTTTATGTCTGGGAGCAAGTTACTTAACCTCCCCAAGCCTGTGTCTGTGAAATGCGGGTA
AATGAATGTAGATGTTTGGCAGCAGCTACTCCTTGTTGAGCTCTCACAGTGAACTCTCCTGCCTCTGCCCTCCTT
CCCCGCCTCCCCTGGTGCCTAGCGTCAGGTCTAGCCACTTCCTCCTGGGCCCCTCTCC**C**TTTTCTGTGGCTGGCT
GCCTGCCCGCCTGGCGCTGGACCTTTCATGTAACGGGAATCAGCATGTATATTCTGGTCTGGTCTGTTTCTACAC
TTAATTTTGTTTCCAGTAGTATTTCCCTGTACCGGCAGAGTTCACAAACACATTTGAAGAGG**CTTTT**TCTCAGGA
TTCTTAACCTTCCCAAAGGAAGTCCCATGGATGGGTTTCTAGAAGTCTATAAATGCTCTGAAATTGTATTTTTCT
GTGGAAAGCATAACTTTCATCTGCTTGTTCGTGCTCAAAAAAGATCATGAATGAATGATTGCATGATTTTATGCC
ATTGTGCTTATACTAAAGGATATGTAGCCCATCTCTTGAGCTGTTAAACTGTTTTGACTACTTTAAATCGTGCAG
CTGTGAGCATCTCTGTAAATTTAGTGTACACATGTATCCCCTGGAGTGGCATTGCCTCGGCAGTGAGCACTTATG
GTTTTATAACTCTCTTCACAGACTCAAATGACTCCAGAAAGCTACACTTCCTGTTGTGAGTATATGATATCCATT
TCCCTACATAGCCACTAACATCAGGTTTTTACAATTTATTTATTTCTTGCTACTTTAAGAAATTTTTGTGGTGA
AATACATATAATAGAAGTTGACTATCTGAATCATTTTTAAGTATACATTCAGTAGTGTTAAGTATGTCGCCATTG
TTGTACAACCAATCTCCAGAA**CTTTT**TCATCTTGCAAAACAAACTCTGTACCCATTAAATAACATTAAACATTCC
ATTCCCTCCAGCCTCAGCAACCCCATTCTACTTTCTGTTTCTGTGAGTTTGACTATTCCAAGCACTTCATATCAG
TTAAATCATGAAGTATTTGTCTGTCTGTGACTGGCTTATTTCTCTGAGCACAGTGTCCTCGAGATGCGTCTATGT
TGTAGCATATGTCAGAATTTCCTTC**CTTTT**TAAAAGATCCAAATAATATTCTTATTTTATAT**CTTTT**TTTTATCC
ATTCATCCATTAGTGGACACTTGGGTTG**CTTTT**GGCTATTGTAAATAATGGTGCTATGTACAAATATCTATATTA
TTGTATTTACAAGTATAATGCTGTAATGTACACACAT**CTTTTT**GAGATCCTACCTTCAGTT**CTTTT**GAGTATATA
GCCAGAAGTGGTATTACTAAATCTTACGATATTTCTATTTTTAATTTATTGAGGAACCACTGTAGTTTTTCATAG
CAACTGCACCATTTTACGTTCTCACCAAGAGTGCACAAGGGTTCCGAGGTTCCCACATCCTCCCCAACACTTGTT
ATTTTCTG**CTTTT**TTTAGATTGCAGCCATCATAGTGGGTGTGAGGTGACATTTCATTGTGGTTTTGATTTGCATT
TCCCTAATGAGGAGTGATGCTGAGCAT**C**TTTTCATATGCTTACTGGTCATTTGTATGTTGTCTTTGGAAAAATGT
CTATTCAAGTCCTTTGACTATTTTAAAAATTGGGTTATTAGAGTTATCGTTGTTGTTGACTTGTAGGAGTTTCTT
TCTATATTCTGGATATTAATCCCCTATCAGATATATGATTTGCAAATATCTTCTCTTATTCCATAAGGTTA**CTTT**
TTCACTTTGTTGATTGTGTTCTTTGATGTATAGAAGTTTTTAGTTTTGAAATAGTCTAATTTATCTGTTTTTA**CT**
TTTGTGGTCTGTG**CTTTT**GGTGTCATATCCAAGAAATCCTTGCCAAATCCAACGTTATAAGGTA**CTTTT**AAGGTA
TTTTAGTTGTCTTAGTCTATATTTCTGTACTCACCTTTCTTTATCCACTCATCAGTTGATGGGCATGTAGGTTGG
TTCCATATCTTTGCAATTCTGAATTGTGCTATGATCAGGTGT**CTTTT**TAGTATAATGATTTACTCTCCTTTGGGT
AGATACCCAGTAGTGGGATTGCTGGATCGAATGGTTTTTATAATTTTCTATTTTACCACAGTTTCTCTCTGCATT
TTTCCTCTTTGACCACTAACCATGTGAAATTCTCATATTGACCTTTATAATGATCATGAACTCTTAGTATCATTG
GGAAGGCCACATTTGCCACTTATGATTGTAAACCTTATCCTCCATTTTTCCTGTTATTGTTGGTGCAAAAAGCAC
CTATTATACCAGGACTTTAAAAATCAGTCTGATAAGTCTTTGATAAGTCTAATAATAATAACTGATAAGTCCATT
GAATTTGCTTCTGATTA**CTTTT**TCTTTAGTAGCTAAACATGTATGTACTCCTATGATTACAATGAACACTCCTCT
CCATTTAAATTAATTATTTACATTGATGAAATAGCAAAATGTTAATGACTAAATACTGTCTTGGTTTTTTCGTTC
CAGGTCAGTCAATATTAACTTCTTATAATTTTCTTTTTTTTCTTTATGTGTGTGTGTGTGTATTTTTTTTTTT
TTAATTTCAATGG**CTTTT**GGGGTACAAATGG**CTTTT**GGTCATATAGATGAATTCTACAGTAGTGAAGTCTGAGAT
TTTACTGCACCGGTCACCTGAGTAGTGTACATTGTACCCAATATGTGGTTTTTTATACCTTGCCCCCCTCTTACC
CTCCCCACTTTGAGTCTCTAGTGTCCATTATGTCACTCTGTATAC**CTTTTT**GTACCCATAAGTTAGCTCTCACTT
ATAAGTGAGAACACACAGTATTTGGTTTTCCATTCCTGAGTTGCTTCACTTAGAATAATATCCTCCAGCTCCATC
CAAAATTGCTGCAAAAAAAAAAAAAAACCACAAACATTATTTTGTT**CTTTT**TTATTGCTAAGTCATATTCCATGGT
GTAGAGATACCACATTTTATTTATCCACTCACTGGTTGATGGGTTGGTTCCACATCTTTGCAATTGTGACTTGTA
CTGCCATCAAGTGTCTTTCTGGTATAATGACTT**C**TTTTCCTTTGGGTAGATACCCAGGAGTGGGATTGCTAGATC
AAATGGTTCTTAACATTTTCTCTCTGGATCTATTTCTGGAAATTTTAGGCTCCAGTTTTTGTTGTTGTTGTTAAT
AAAATGCAATGGAATGTAATGATCATCA**C**TTTTCATTATGCTTTAAAATCTGGTAAATGGAGGCTAGAACACTCC
TGTAAGGCAAGAATATTCTCTCTGTTGGAACTCAAATACACAGAACTGGGTAAATCTCAATCTTAATCTTTGATT
CAGGACACAACATGGCTCT**CTTTT**ACTTGCTTTCTTTAATTGTTTTTTAATAATGTGGTAAGCATTTCTGAATCT
CCTATCCAATACAAAAACTAGGACAATACAGACAGTAACTCCTATGGTTACAATGAACACTCCTCTCCACTTAAA
TTAATTATTTACACTGATGAAATTGAAATAGCAAAATTTTAATGACTAAATACTGTCTTTGATTTTTTGTTCCAG
GTCTGTCAATATTAACTTCTTATAATTTTCTTTTTTTTTCTTTATGTGTGTGTGTGTGTGTATATATATATAT
TTAATTTCAATGG**CTTTT**GGGGTACAAATGG**CTTTT**GGTCATATATATGAGTTCTACAGTAGTGAAGTCTGAGAT
TTTACTACACCTTCCACTTATGTGGTCCCACACCACCCGCCTCCCCTGCCGCCTCCTGCCACCCCCTAGGCCAAG
GTAATAATCATCCTGAATCCTGGGTTTATCTCTCACTTGCTTTCTTTTCATATAATTTTGCAAAAGAATCTGATC
TAAATGTGTTTTTCAGAGTATATATTTATATTTTAGCTGTTCTTAGAGAAAATTTATTATTTTGCATGTAATCTT

ATGGAACATTCTCATTTAATACCATGGTAAGATTCAGCCCTTGCCCAGGGGATAGTTCATTTAGTTTGTTTACTG
GATAGAGCTCATCATGTGACTATACCTCAGTTAGTTTATCAGTTCTCCCATCCATGGTGACTAGGTTGCCTCTCA
GCCTCTCAACAACACTGTTTCTCAGTGTCCTTGTAGAAGTGATATGTGGGTGTTTTCTCCTTACACAGAGTTGAA
AGGTGACGACAACAACGTTGGCACTACCAATCCCCCACCCTCCAGAGGGGTAACCAGTGTTACCAGTTTGCTGTG
TTTCCTGCTACACCTCGCCTTATTCACTTCCATTTGTATCTGAAAAACGTGTTGCATGGTTT**CTTTT**CTATAGAA
GTGGTAAAATGCTATTGTGTCCTGTACATTATTGATTA**CTTTT**TTTCATTTAACAGTAGGGAGATGCCTGGGAGT
ACACAGAGAACTGCCCTCATTGTTTTCAACTTCTGCACTGTATGTCTGTGAGTTTAGCCATTCTGCTGTTAATGG
AAATTTACAGTATTCTAAT**CTTTT**GATATTACAAACAGTTCTGTGCGATCATCGTCATACACAACCCCTTGTGCA
CAATGCATGAGTGTTTCTCAGGGTAGGTACCAAGAAGTGAAATTCCTGGGTCATAGGGCGTGAGTCCGACATTTT
TCTCCATTCTGCCCTGTTGCCCTCCAGAGTGGGTGTCCAGCTTTGCATACCTAAGTATGAGAGTATCTGTTGTTC
ATATCCTCTACGACGCTCCATATATGAAACTTAAGTTTCTGCTAGTTGCCATCTTTGATCTATCATGTATGCAGT
GACCTACTAAGACTGTAATTGGTACAGTAGATTCTTGTCATCTGTGTGTGAATTTAGCATTCATGGGCTTAATGC
TGACAAGGCCCCCAGGGTCCAAGACATATAATCATGTATAATTTTGTCAAGGTATAATTTTTTAAATTG**CTTTT**G
TCATGTGTCTGCTGGTGATGCCCAACCCAGTGCTCTGCACCCAGGTCACACTGTGGCTTTGTCCTCTGCTTATGC
CTGCATTGCAGCAACTGTCCTGAAGAGACCAAAATTATGCAGATTTAGGTAAGTCCATGGCTAATGTTATTATAT
TATGTGCTATTGTAATGGATGGGGCTGTGGAGTGTATGAATTTATAAATCACTGGTCTTGTAATTAAAATTCAAA
CACTATAGAAAAAGGCCATGTAGAAGATAAAAGTTCCTCTATAATCCCGGACCCCTAAGATAACTACTAATGACA
ACTTCATTTATATTCCTTCAGACATTTTCTGGCTGTGGATGTACTAAAATGTATCCTATTATTCTCTGCCCTAAA
ATGGAATCATACAAGGTGTACTGTTATTTTTATGGCTCTATAACATGTCATATTGTACGTGTTGGTATGGTCATT
TTAACCATTTTTCTAGTGATGGCTTTGAGGTTATTTGCAGTTTCCTAGCCATCTCAAAGTGTGCTGCGGGGATCT
CTTTTGCATCCCTCTGGGTGCAGAGCTGAGGCACCCAGAGGCAGTGTCCAGAGGAGGCAGCATCTGTAGGTGTCT
TCACCTGCTCTGGCTCTTGGCACATCTGGTTGGTGACACTGTTTTGTGAGATGGGTTGAAAGCACGTGCTGCCAA
AATAGAATAATGTTGGTCCTCTCCTCATGTGCCGTGGAACTGGGGTAAAACTGCGTAGTGGCTGCAGCTGCCTGT
CCATACCGGAATCGAGTATAACACGGTGCCTGGCTTAGCACAAAACAGTAGTGGGTCCTGCAGGCCCCAGAGTCT
AATTCCTGGTATTCTTTCCCCTACACAGATTAAATAAACCAAAAACAAACTATTCTAGGAAAGCGTCTGTGACAT
TTGTAAAAAGTGGTATTTAATGAT**CTTTT**ATTCACTTGTCTGTTTAGTTTGTTGAAATCTTAAGTGGCATCCTGG
TCTGGGAAGGAGTGCTGTCTGCGCCTGCCCTCCGCTGGGCACAGCGTGGCTGCTTCAGGGGCTAAGCACACACTT
TCTGTCTTCTAAAGGGCCGCCACATGCCAGGAGCTCAGGTGTGAGCCCGGCTCTGGCTCTTACCTCATAGGGTCA
CTCATAGGGGCACAGGGAGCAGAACATTGTACACAGCGAGGCACCACCCGGCTTGGCATCTGCCTCGGTGGACTT
ACTACCTCTAGAAGGAAATACCTGAGTTCCTCTGGCCTCAGCTCCTAGAGTGACTGGTGTGCTGTCCCTGTTACT
CTTCTGTCAAGGTGACAACTGTGTGACCCATCATCTGTGTGTCAAAGCAAGGCCCTGCCTGGGCCTCTGCTCCTG
TGCTGACCCCAAAGGCAAATGCTTTGCTAGTTTCCTTCCAGTTAATTTCACCTATGAATAGATGTGTGAAAACTG
TTCAAAGCCATACCTGCACATGTTTGAACTTCAAACCCTGTGGGTGATTCAGTGGCATCTTTCTCTAACCCCCAG
CCTCCCTTCCCACAGAGGCCACCGTCATGGCCAGTTGCTGCAGTTTCTTTCCAGAGAACCTGTGTATGTGTAAAG
CTGTACAGGCGTGGGTACACCACACAGCCTGTCTTGCACTGTGGACTGTTGAGTTACTAGTACATCTAGGTAAGC
ACCGCATATCTGTATTCATGTCTGCCTTGGT**CTTTT**CAACATCTGTGTGGTAGCCGTGTTTGAATTACCCATTCC
CTTTTTGGGGAACCATTAAGTTGTTTCAGCAATTTTTACTGTAGATAAGGCTATACCGCATATCTGTGTACATGG
GTTTTTATGTACATGGGCAAGTATATCTGTGAGAGAAAAGTTTCCTCAGGAGGAATTCTGGGCACAGCATGTGTA
AATTTCTAAATATGATGGACACCCCCAGCTTCCACCTCAAGGAGGTTGGTCCCATTGACATTTCCCCACACCTTC
ACCCAGGCTGTGCCCTTAAACTTGGTTATTTGTCAATGTGAGAAGTGGAAAATAGTATTTAATTGTAGTTTGGAT
TTGTATTTCTATTGGGTTGTATACTTACTGATTAATAATAAGAGCTCTTTACATATTAAGGAAATTAACC**CTTTT**
CAAATACATTCCTATTTCTCACTAATCTTTAAGTTTTATTGTAATATTTTGCTCTTTAGTTTATATATATATGTA
TATATATATATATGTATATATATATATATACATATATATATACATATATATATACTAATTTT**CTTTT**ATGGTTCC
TGGATTTTGTGAGTAGTTTGAAAAGGCTAATCCAGCTGAAGATTTTGTTGTTGTTGTTAAACCCCATGTTTTCTC
CTAACTCTTTTTATTTTTATTTTGGAGGACTCTATCTAGACTTAATTTTAGCATAACAAGTGACAGGGTTAGTTA
GCCTGTTGTCCTTACACCATTTTCTGGCTAATACAGCTATTAACTATTGATCTGTCTATTCACGTGCCAGTTCCT
AATGGTTTTACATAGTGTAATCTGCACTTCAAAATAGCGAAGGGAAGCCCTACCTCATTATTCTA**CTTTT**CCAGA
ATTCTCCTGGCTATTCCAGGCTGCATGTTTACCTTAACCTTCCCTGTGATGTCTTCATGCCGTTGTCTTCTTATG
CAAGAATAAGGTACGTCTTTCCATCCACTCACGTCTATTTAATTTGACTTTGCATTACACAGAAAGCTGGTCTTG
GTCTGTCTACCTCGGCATCTAGTTGTCCTCACTGCCCCCTAGCCGACCCCACCCCATCTGACTGACTACCCCATC
ACAGAGTA**CTTTT**ATTTACGTTTTGCTCTGCCTAATGGTTACTTGATACTGTCACGCCGACAGTGTCCAGTTCAG
TGGTCTTTGCAGTTGAAATGCTCCCGTACACACTGTCTTGTTAAAAATGCCAGTAAGTTCATACAAACCCAGCTT
GCACCCAAGGTCACATTCAGAGAGCGTAGGGCTGGGATGGGTTGTTTTCCAAGCTTCTGCCACTGTGTGGCTAGC
TCTTCCCACTGGGAAGTTCTGTGTACCCGGAATGTCGGAGTGGAGTCCTGTTCTAGTGTCCAGCACCTGACCCTG
TGCCCAACCCCTCAACAGCCTATTCCTGCTGTCCACAGCCTGCTGGAA**CTTTT**TACAAAATATGTTGCCATGCTG
GACCCTGGGCACTGGACATAAGCCCCCTGGCAGC**CTTTT**TCATGTCACCCAAAGGGGTAATTGTCCTACTGGTGG
TCTGTAAGATGAGTTAGGGTGACTTGCTAATAGACATTGTAAATCTTAATATTTATGTATGTATTTTATTATTAC
CGGTTTTCCATTTATGATGGTAATATTGTTTCTTCTAAGAATATTTATTTTTCCTTCTAAATATTGAGATAAAAT
TCATG**CTTTT**GAAATGTTCTATTCAGTGG**CTTTT**AGTATATTTGCTATGTTGTGCAACCATCGACACTATCCATT
TCTAGAA**CTTTT**TCGTCATCCCAAACAGACGCTCTGTATTCATAAAAAAATAACTTCCTACCTGTCTCTCCCCCT
AGTCTTTGGTAACCTTTGTTATACTGGTAAACTTTGTTGTGCTCTCTGTCTGTGTGAATTTGCCTATTCTAGGGG
CCTCATATAAGTGTAATCATACAGTATTTGT**CTTTT**TGGGTCTGTCTGATTTCACTTAGCGGGTTTTCAGGGTTC

ATTCATGTTGCAGCATATAACAGTACTGCGTTCCTTTTTCTGGCTGAATAATATTCCACTGTATGGATAGACCCC
ATTTTGTTTATTCACACATCATTTGGACATTTGGATTATTTCTGGTTTTTGGCTATTATGAACAATGGTGCTATG
AACAGTTGCGTACAAGTTTTTGTGTGAACATATGTTTTCAATTCTCTCATTATATACCTAGGAGTAGAATTACTG
GGTCATATGGTAACTGTATATTTTTGAGGAACTGCCAAACTATTTTCCCACGTCCATGCACCATTTCACATTCCC
ACCAGTAAGTAAGAGGGTTCCAATTTCTGCGCATTCTTGCCAACACTAGTTATTATCTGACTTTCTGGTTATAAT
CATTCTAATGAGTGTGAAGTAGCCTCTGGTGTCATTTGGATTTGCATTTCTCTGATGAGTGATGCTATCAAGCAC
CTTTGCTGGTGCTGTTGGCCATATGTGTATGTTCCCTGGAGAAGTGTCTGTGCTGAGCCTTGGCCCACTTTTTAA
TTAGGCGTTTGTCTTTTTATTACTGAGTTGTAAGAGTTCTTTATATATTCTGGATTCTAGACCCTTATCAGATAC
ATGGTTTGCAAATATTTTCTCCCATTCTGTGGGTTGTGTTTTCACTTTATCGATAATGTCCTTAGACATATAATA
AATTTGTATTTTAAAAGTGACTTGATTTGGCTGTGCAAGGTGGCTCACGCTTGTAATCCCAGCACTTTGGGAGAC
TGAGGTGGGTGGATCATATGAGGAGGCTAGGAGTTCGAGGTCAGCCTGGCCAGCATAGCGAAAACTTGTCTCTAC
TAAAAATACAAAAATTAGTCAGGCATGGTGGTGCACGTCTGTAATACCAGCTTCTCAGGAGGCTGAGGCACGAGG
ATCACTTGAACCCAGGAGGAGGAGGTTGCAGTGAGCTGAGATCATGCCAGGGCAACAGAATGAGACTTTGTTTAA
AAAAAAAAAAAAGTGACTTGATTTAAGGGAAAAAATGACTGGCTATATTCAGTCAGATATGGCAAAAAGTCTCAA
GGTGTTAATGTGAATGATTAAGGTCTTGGGGGGGGTGTCCCCTATCAGACTACAGGTGTTTAGAGGCACAGAAAA
AGGTGCAGTTGGGTTCTTAATGTGAAATGATGAGAAGCACAACTCCAGTGTGTCTCTTTGTGTAGAATGTCAGCA
GACACCCCCTGCTAGATGTGCTGGATCATGGGAAAGCATTTCCATTTGTTACTAGATTGTTCAGAAGTTTTAATT
TATGATGGGTGTGGTGGCTCATGCCTGTAGTCCCAGCACTGTGGGAGGCTGAGGCAGGAGGATCATCTGAGGCCA
AGAGTTCAAGATCAGCCTGGGCAACATAGTGATACCCTATCTCTTAAAAAAGAAGAAGTTTTTAAATTTGAAATA
ATAATAGGTACTGGATTTATGCAAATGTCTTTTCTGCGTCTTTTGAGATGAGTATCAGGTTTTTTTTTTTCCTTT
TATCATCTGATGATGAACTTAATGTTTCCATTTGTATTAATGGAATACTAAGTCCCTCTGTGATTTCTGAACCAA
GCTATTCCTAGGCCTGAGTTTTATTTTGTTGACACAGAAATAAATTAGAAGGCCAAGCGTGGTGGCATGTGCCTG
TAGTCCTAGTTGCTGAGGTAAGAGGATTGCTTGAGCCCAGGAGTTCAAGGCTGCAGCAAGCTTTGATTGCGCCAC
TGCACTCCAGCCTTGGCGACAGACTAAGACGCTGTCTCAAAAAAAAACAAAAACGACAAAAAAAAAACAAAACAG
AAAAAATAAACTAAGGCAATGACAGTCCCTGGCAAATGCTGGGAGGGAGGCAGCAGTGGTCAGGGAAGGTAACCC
TGAAGCAGGACTTGTAAAGCAAATAAGATTGGGAGGCCAAGGTGGGTGGATCACGAGGTCAGGAGTTCGAGACCA
GCCTGGCCAACATAGTGAAACCCCGTCTTTACTAAAAATACAAAAAAATTAGCCAGGTGTGGTGGTGGGTGCCTG
TAGTCCCAGCTACTTGGGAGGCTGAGGCAGGAGAATCTCGAACCCAGGAGGCGGAGGTTACAGTCAGCTGAGACC
GCACCATTGCACTCCAGCCTGGGTGACAGAGCAAGATTCCGTCTCAAAAAAAAAAAAAAAAAAAAAAACCAAGAAG
AAAAGGAATGAATTAGAACTTCTTCTGCTTGGACTTAAGGGCATCATCAGGCAGGTTTTGGGTAGGATAGCAGGG
GAGGCAGAGACATAGTCGGGGTCAGTGGTCATGAGTGTGGCTTTGAGCCCAAAAACTTGGTTTCTGTTCCCTACT
TTGCCACTCAGTAGTGCATGACTTTGGCCAAATTTCTTAAATTCATGAAGCAAGTTTCCGGGTGAATGAAATGGG
GATAAAAATAGTGTTCAAACCTATCCGTTGGTTTGTGTGAAACTGAAATGAATAGTATCGTGCAGGTACTTGTGA
GCAAGGGGAGCTGCTGTTTCCTGTCCCTTTATGATGGGAAATATCTAGACAAGTTCCCAACCCTCTGCACTGCAG
GCTGCATGGCACGGAGGGTCTTGTAACACCAGCTGGGGCTGGCCTTCTTTTAGGAGCTTCAGTGGTTCTGAAAAC
TTTTATTTGTTTGTTTGTTTTAGTAGATGTGGGGTCTTTCTGTGTTGCCCGGACTGGTCTCAAACTTCTGGACTC
AAGTGATCCTCCCCCGCTCAACCTCCCAAAGTGTTGGGATTACAGGTGTGAGCCACTGTGCCCAGCCTTGAAAAC
TTTTTCAGGTTCTTCCAGGGTTACTGGGCTATTAAATATTTCTATTTCATTATAAGTCAGTTTTTCAAAGTTATA
TTATCTTAATTACCTTTTTTATATGTATTAGTGTAGAGTAGCATTTTATATTTTGATATCCTCCTTATGCATAGT
TTTTCACTTTTTATTCCTAGTTTTTCGTTTTTAATAAGACTTTCAAGAAATTTATTTTATTGGCCTTTTGAAAAA
AGCAGCTTTAGATAAAGTAAGCAGTTCTGCTTTCATTTTATAATTTATTTCTACTTTTGTTTCATTAATCTTTTC
CTCCGGCATGCCTTGGATTTTGTTGTGTTACTCTTTTTCTAGAGGCTCGCATTGTGTGTCTGGTTCACTTATGAT
CACGCTTGCCTACTTTTAAGAATGGAAGAGGGGAGGTGGAGGGTGGCTGCACAGTCGAGGGTGTGAGGCAGTCTT
GCTCTAGCCCCACCATGCCCTCAGCCCGCTGTGGCCACGCTGGTTCCTCAATTGCTGGGGCGTGCAGTGTCTGTA
AGGGAGGCTACTGATGCCATCCGAGGAAGATGTAAGGTTTCGTGTGGGCAGCGAGAGCCTAGCAGGCATGTGGGG
TGCCCAGCAAAGGGTAACAGTGGACAGTTGTTGCCTCATTCCACAGAGTTTTGATTTTTTTTTTTTTTTTAATGG
TCACTCCATCAACATCCCCCATGGCCAGAGCCTGAGCTGGTCCCCAGAGACACAGGCATTCAGCTGACAGCCTCG
CCTTCACGCTGCTGCTGTTCTCATGGGGGACAGGCCTCAGGTGGCAATGCACAAATCATTAGTTAAGGGCAGTTG
TGACAGTTACCAAGGAGTGTAGTCCCCCGCCCCCCGCCCAGTGAAAACAGCCCTAACCAGGGGTGGGGACCTTTG
GGCTCTGACCCGAAGGGTAGGAGAAGCTGGAAGGACAGCATTCCTGTCTGCGAAGGCAGGAGCAAAGCTGCCAGG
CTATGAAGGAAATGGCTGGAGCCTGAAGTCATGCAAGCTGGGGCTGGCAGGGACAGGGCCAACTTCCAGGCCTGG
GGGCCACCATGAGGATTCAGGACGTGACCCCCAGGGCACATGAAGGCCTTCCATCTGTATTTAAGAAAAGACTTT
ATCAGACGAGTATGGTGGCTCACGCCTGAATCTTAGCACTTTGGGAGGCTGAGGCAGGTGGATCACGAGGTCAGG
AGTTCAATACCAGCCTGGCCAATATGGTAAAACCCCATCTCTACTAAAACTACAAAAATTAGCCAGGCATGGTGG
CGCACGCCTGTAGTCCCAGCTACTCGGGAGGCTGAGGCAGAAGAATCACTTGAACCCGGGAGGTGGAGGTTACAG
TGAGCCAAGATCGCGCCACTACACTCCAGCCTGGGTGACAGAGTGAGACTCCGTCTCAAAAAAACCAAAAGACTT
TATCTTATTTCCTATATGTTTGTGGTTTCAGTCCTGATGTATAATTTGACCCTAGTTAGAATGGTTATCTGAGGA
AGTGGCCTGTACGATTTCTGCTTTTTTAAATGTGTGGCTCCCTTTCTTCATTGATTAACGTATGATTATTTTTAT
AAATGTTCCATGGCAGTGGGAAGGGATTCTCTGTCACATTCCACATCTGGATCAGTTCCTCCCCATTTTGTTGGT
CAAATCCGATCTGCCATATCCTGTGTAATGACAAGTGAGTTGCATTCTCACCGTCACTCCTGGGGTCTCTCCGCT
TCCCCTGAGCTGGCTCAGCAGTCTGCTCCATGTGTTTTGATGCAGGGTGACCCATTGGTATTCCCGACACTAACG
CCCCCGTCTGTGGACTGCTTGCTGCTTGGGCTTCACTGTGTCTGGTGTTGACAGTGCAGACCTAAAGGTGTGCAC

ACATGTGCACACACACTCCGCTGTCTTCTTGTTTGCACTGGACTTAAATATCTATGAGGGTTATTTTCAACTGCT
GAATTTGGAATGATTTTTATATCTTTTCTGCTTTCTGCCCATGTACATGTGTTTATTTTACACTGTTGTGATTGG
TAGTTACTATGTGGGGACACAATTACTTGGGCTGAAATAATCCACCTGTTGTGGTTGGGGTCCTCTGGGGCATTC
CAGGGTGAGAGGTTGTCACTGCCACCTGGGCCATGTGGGCCGGCACCAGCATTTTGTGGTTACGAATTCTACAGT
CACAAATATCTTTGGGCAAATCCCCTTCTATACCTCAAGGCAG**CTTTT**GGTTTGCAACCCCACTGGCCAGAGGGA
AGGGCCAGTCACTTGGCTCTCTCACTGCCCTGCGCCCCAGATGGTTCTAGGGCTGCTGTTTTCCCTTGGCCCTGC
CAACACCACTGTTTTTACTTCTGCTCATTGGCTGAGTGCAGTGGTTCCTGGAAGCCAGTGGCACGTTTCCCCGCG
TAGCTCGCTTATCCCACAGCACACACCCAAGGGTTCTGTTGCTAACACGCTGAATTAATTCTTTGCTCATCTTAC
AGAGTGTGTTTTGACTGCCCCCATTTCTGAGGCCTTGTAAGGCCAGAGCTTTGTTGCTTCATCGGCAGGTTGGGA
CTTAGATGGCCGTGAATGTTTCCTCTCTGCTGCTGCAGTAAGTAAGTGCCCGCACCATAGTGTGTTTGGAGGCTG
AAGTTGAAGCGAGGCTGTGAGGGGAGATGGACGTGTGAGGAGGGATGATGGGGCTTGAGCAAAGTGGGGGAGGGG
GCAAAGGCAGTTGGCCCAACACATTCCCCACCCCTTTGAGAGGTCTGAGGCCTGCAGACCTGGCTCGGAGCCCAC
CTGGTAGTCCTCAGACTGTGTGTGTGTGTGTGTGTGTGTGTGTGTGTGTGTGTGTGTGTGTGTGTGTGTGTGTGT
AAAAGAGAGAAGTTGTGGAGAAATGGGGGGCTGATTCTGCTCAGATTCATCAGGATGAGTAGAAGGCACCCAGCT
CTCACCCTGGCCTGACATGTGTGTCCCTGAGCAGGTTACAGTCCTCTCTGAGCCTCTGCTTCCCATCTGGACCCT
GCTGGGCAGGGCTTCTGAGCTCCTTAGCACTAGCAGGAGGGGCTCCAGGGGCCCTCCCTCCATGGCAGCCAGGAC
AGGACTCTCAAATGAGGACAGCAGAGCTCGTGGGGGGCTCCCACGGACCCGCCGTGGGCCCAGGGGAGGCAGAGC
CTGAGCCAACAGCAGTGGTGCTGTGGACCGTGGATCCTGAGGGTGGCCTGGGGCAAGTACCGGCTGAGGGTCCAG
GTGGGCTTTGTGTACCTTTGGGTCCTGGGGCCCTGGTGACTTGGACTCCAGGTTAGAGTCAAGTGACAGGAGAAA
GGCTGGTGGGGCCCTGTGCTTCCGACTTCATTTCGAGTGATGGCAGTTCCCAGGAAGGAATCCACAGCTGACGGT
GGCTGACAGATCAGAGAATGGAAGGCGAGGCAGGCGGGCGTCTGCGTGACCTCAGGTGCTTGGGGCCCAGCAGAC
CCAGAGAACCATTTCCACTAGGCCAGGGTGCCGGAAGTGTCCACAGGTCTTAGATTCCCTGTTCAGATGAAAAGA
TTTGTGCCTTTAATGATAAAAGTGATCTGCATAGAGTCAAAAATTCAAGCCATGGGTATAAAATGCAAGTAAAAT
CCCTGCCCTCACCTATCCCACCCTACTACACAGAGATGTCCTCTCGAGTTTCCTAGACTCACTCTGGAAATTTCT
GTATACACACAGAAGCTTGTGCCTCTGCTCGTGAAGGCAGAGGGAGGGAGAGCTGAAGGGCCAGCACCTTCTCAC
CTGTGGGCCCCCTCAGTGCTCGGTCCCAGAGCATGCAGGACTGTGCCTCGTGTTCAGTTTGCTGGTCTGACTTCA
TGCTCCTTGGGCAGGATATGCATGTGCCATGCTAGGAGACATGTGGATGTGAAGCTGGGGGACAATGTCCCCTGG
CTATGCCTTTACAAGGGAAGTAAGGAAGGTAGGAGGTGAGCCTGGGAGGGAGGGAGGGAGGCGCGGAGCCGCCGC
AGGTGTTT**CTTTT**ACTGAGTGCAGCCCATGGCCGCACTCAGGTTTTG**C**TTTTCACCTTCCCATCTGTGAAAGAGT
GAGCAGGAAAAAGCAAAA
